# Two-Stage Self-Supervised Meta-Learning for Few-Shot Cross-Condition Fault Diagnosis of Air Conditioning Compressors

**DOI:** 10.3390/s26216802

**Published:** 2026-10-28

**Authors:** Tao Feng, Jiewen Wen, Jing Wang, Kun Chen, Haonan Guo

**Affiliations:** School of Computer and Artificial Intelligence, Liangxiang Campus, Beijing Technology and Business University, Beijing 102488, China; fengt@btbu.edu.cn (T.F.); 2431062217@st.btbu.edu.cn (J.W.); 2431062206@st.btbu.edu.cn (K.C.); 2431062194@st.btbu.edu.cn (H.G.)

**Keywords:** fault diagnosis, few-shot learning, meta-learning, self-supervised learning, prototypical networks

## Abstract

**Highlights:**

**What are the main findings?**

- A two-stage self-supervised meta-learning (TS-SSML) framework is developed for the few-shot cross-condition fault diagnosis of vibration-sensor-monitored air conditioning compressors, which combines NT-Xent contrastive pre-training and attention-weighted prototypical learning to extract fault representations transferable across operating conditions and improve classification discriminability.
- The proposed method achieves a 96.50% accuracy on the 10-way cross-condition transfer task and 94.10% accuracy on the 6 → 4-way incremental few-shot novel-class detection task under the 5-shot setting; it outperforms standard prototypical networks and cuts episodic meta-training time by 93.75% (Stage 2 few-shot adaptation only) compared with SCM-FD while maintaining competitive diagnostic accuracy. All results are obtained on a single self-collected compressor dataset under two controlled operating conditions; generalization to a wider range of rotating machinery is posed as a hypothesis to be validated on public benchmarks, not as a demonstrated fact.

**What are the implications of the main findings?**

- This framework effectively lowers the reliance on large-scale labeled fault samples, providing a low-cost and easily deployable solution for industrial vibration-sensor-based compressor quality inspection scenarios with scarce labeled data.
- The study provides a practical technical reference for the predictive maintenance of refrigeration compressors; its generalization to a wider range of rotating machinery remains a hypothesis to be validated on public benchmarks and other types of equipment, and is left for future research.

**Abstract:**

The air conditioning compressor serves as the primary power unit in refrigeration systems. Vibration data acquired from vibration sensors can be used to implement fault diagnosis, and reliable fault diagnosis is critical for ensuring safe and stable system operation. However, existing data-driven approaches face two fundamental bottlenecks: the prohibitive cost of acquiring large-scale labeled fault samples in industrial settings, and the degradation of model generalization caused by a domain shift in vibration signals across different operating conditions (e.g., high-pressure vs. low-pressure modes). To address these challenges, this paper proposes a Two-Stage Self-Supervised Meta-Learning (TS-SSML) framework. In Stage 1, we perform self-supervised contrastive pre-training on large-scale unlabeled vibration signals using the Normalized Temperature-Scaled Cross-Entropy (NT-Xent) loss, enabling the encoder to learn fault representations that are transferable across operating conditions. In Stage 2, the pre-trained encoder is frozen, and an Attention-Weighted Prototypical Network (AWPN) is trained via episodic meta-learning to dynamically assess the contribution of each support sample to class prototypes, thereby enhancing discriminability in few-shot classification. We conduct systematic evaluations on a self-collected AC compressor vibration dataset across two tasks: cross-condition transfer (Task T1, 10-way) and incremental few-shot novel-class detection (Task T2, 6-known-to-4-novel). Experimental results demonstrate that the proposed method achieves a 96.50% (T1) and 94.10% (T2) classification accuracy under the 5-shot setting, outperforming standard Prototypical Networks by 1.20% and 4.92%, respectively. Compared to Sparse Coding-based Fault Diagnosis (SCM-FD), our method reduces the episodic meta-training time by 93.75% (Stage 2 few-shot adaptation only) while achieving superior accuracy. These results highlight the substantial advantages of the proposed framework in industrial fault diagnosis scenarios with scarce labeled data.

## 1. Introduction

Rotating machinery acts as the critical power and execution component in intelligent manufacturing, petrochemical engineering, aerospace, and other key industrial sectors. Its operational stability and health status directly determine the safety, reliability, and service life of the whole equipment system [1,2,3]. Driven by alternating loads, material fatigue, assembly errors, and severe working conditions, key components such as bearings and gears are prone to incipient minor faults, which will gradually evolve into critical failures and eventually cause unplanned shutdowns, substantial economic losses, and even catastrophic accidents [4,5,6]. Therefore, achieving the accurate, rapid, and early fault diagnosis for rotating machinery is of great engineering value and theoretical significance to guarantee continuous production, reduce maintenance costs, and avoid major safety risks.

In recent years, data-driven intelligent fault diagnosis methods have developed rapidly and achieved remarkable progress in feature extraction and pattern recognition [7,8,9]. Traditional machine-learning methods, including support vector machine (SVM), random forest (RF), and extreme learning machine (ELM), rely on manually designed statistical features, frequency-domain features, and time-frequency features. These methods heavily depend on expert experience in signal processing and exhibit weak generalization under complex and variable working conditions [10,11,12]. Benefiting from the advancement of deep learning, the convolutional neural network (CNN), the recurrent neural network (RNN), the autoencoder (AE), and other architectures have been widely applied to fault diagnosis. Such models can automatically learn robust feature representations from raw vibration signals and significantly improve diagnostic accuracy [13,14,15]. Nevertheless, these methods generally require massive labeled samples for supervised training and assume that training and test data are independently and identically distributed, which is only valid in ideal laboratory environments.

In practical industrial scenarios, the operating conditions of mechanical equipment are changeable, the fault modes are diverse and complex, and the acquisition of labeled fault samples is extremely difficult and costly [16,17,18]. In most cases, only a handful of labeled fault samples are available, and even novel unknown fault types that never appear in the training stage may emerge. As a result, the performance of traditional deep learning diagnosis models degrades sharply, which can hardly meet the requirements of engineering applications [19,20,21]. To tackle the few-shot fault diagnosis problem, meta-learning has attracted extensive attention owing to its capability of learning general learning strategies from massive tasks and rapidly adapting to new tasks with limited labeled samples. As a representative metric-based meta-learning model, the prototype network (ProtoNet) generates class prototypes by averaging support set features and classifies query samples by measuring the distance to prototypes. It features a simple structure, fast inference, and strong adaptability, making it suitable for few-shot fault diagnosis tasks.

However, the existing few-shot fault diagnosis methods based on the prototype network still suffer from three critical limitations. First, most models are trained from scratch on small-scale task datasets, lacking the pre-learning of universal fault characteristics from massive unlabeled vibration data, leading to the insufficient feature extraction ability and weak robustness of the backbone network. Second, the conventional prototype generation adopts simple average pooling, which treats all support set samples equally and ignores the discrepancy in sample importance. This results in blurred class prototypes and a reduced classification accuracy, especially under noise interference and sample imbalance. Third, the transferability of pre-trained feature representations to the downstream few-shot classification stage under a condition shift is insufficiently considered. The domain shift problem reduces the transferability of pre-trained features, making it difficult to fully exploit the advantages of the two-stage framework. These issues restrict the performance of few-shot fault diagnosis models in identifying novel unknown faults and adapting to complex and variable working conditions.

To overcome the aforementioned challenges, this paper proposes a two-stage few-shot mechanical fault diagnosis method based on self-supervised pre-training and the improved prototype network. In the pre-training stage, a contrastive learning task tailored for vibration signals is constructed to conduct self-supervised pre-training on massive unlabeled data, enabling the encoder to learn robust and universal fault-related features without manual annotation. In the meta-learning stage, the pre-trained encoder is frozen as the backbone network, and an improved prototype network is built. By introducing an attention-weighted prototype computing strategy, the quality of class prototypes is enhanced, allowing the model to rapidly and accurately identify novel unknown faults with only a few labeled samples. The main contributions of this paper are summarized as follows:

1. A two-stage “self-supervised pre-training + meta-learning” framework is proposed for few-shot unknown fault diagnosis. The vibration-specific contrastive pre-training enhances the feature extraction ability and feature transferability of the backbone network, and the improved prototype-network-based meta-learning enables fast adaptation to new few-shot tasks.
2. An attention-weighted prototype computing module is designed to dynamically assign weights to support set samples, optimizing the generation of class prototypes, strengthening feature discriminability, and effectively improving few-shot classification accuracy.
3. A systematic validation strategy of “overall comparison—component ablation—robustness test—visual analysis” is adopted. Extensive experimental results on the self-collected compressor fault dataset demonstrate that the proposed method outperforms baseline models in few-shot diagnostic accuracy, anti-noise robustness, and generalization to unknown faults.

We make the positioning of the above contributions explicit: contrastive self-supervised pre-training (NT-Xent) and prototypical meta-learning are established techniques, and the contribution of this work does not lie in any single component. Rather, the contribution is the task-specific combination of the two (whose individual contributions are additive, as demonstrated by the ablation study in Section 4.3.2): (i) a physics-motivated augmentation suite tailored to one-dimensional vibration signals (Equations (2)–(5)); (ii) a two-stage coupling design in which the pre-trained encoder is frozen, so that the few-shot stage trains only a lightweight attention module—which also yields the low episodic meta-training cost; and (iii) a systematic cross-condition and novel-class evaluation protocol on an industrial compressor platform.

The remainder of this paper is structured as follows. Section 2 reviews the related work of self-supervised learning and meta-learning in the field of fault diagnosis. Section 3 elaborates the framework, self-supervised pre-training, and the improved prototype network of the proposed method in detail. Section 4 conducts experimental verification, a comparative analysis, and an ablation study. Section 5 concludes the whole paper and prospects future research directions.

## 2. Related Work

This section provides a systematic review and discussion of the current state of research on two key technologies in the field of machine fault diagnosis: self-supervised learning and meta-learning. This chapter aims to clarify the technological evolution from “learning general features from unlabeled data” to “rapidly adapting to new tasks under few-shot conditions,” and to thoroughly analyze the limitations of existing prototype-network-based methods. This provides a clear theoretical background and innovative positioning for the two-stage method proposed in this paper, which combines self-supervised pre-training with an improved prototype network.

A. Self-Supervised Learning in Fault Diagnosis

Traditional deep-learning-based fault diagnosis methods rely on two idealized assumptions: “training and test data are independently and identically distributed” and “sufficient labeled data is available.” These assumptions are difficult to satisfy in real-world industrial scenarios where label acquisition is challenging and operating conditions are highly variable [1]. To break the reliance on labeled data, self-supervised learning constructs supervision signals from unlabeled data itself by designing a pre-training prediction task, making it an effective approach for learning general feature representations from massive amounts of unlabeled monitoring data.

Based on the design principles of the pre-training tasks, the relevant research can be broadly categorized into two types: generative and contrastive.

Generative self-supervised learning: These methods learn features by reconstructing or predicting corrupted input data. In fault diagnosis, commonly used techniques include denoising autoencoders and masked signal prediction. For example, by reconstructing segments of vibration signals that have been randomly masked, the model is forced to learn the local structure and contextual information of the signal. While these methods help models capture fine-grained signal features, their reconstruction objectives may lead the model to learn irrelevant noise patterns under strong noise interference, and there are challenges in aligning the task difficulty with the ultimate diagnostic goal.

Contrastive Self-Supervised Learning: These methods construct pairs of positive and negative samples to bring similar samples closer together and push dissimilar samples further apart in the feature space, thereby learning discriminative features that are robust to data augmentation transformations. General contrastive learning frameworks such as MoCo [22] have been successfully applied to vibration signal analysis [23]. For instance, some studies have combined contrastive learning with specific data augmentation strategies to learn features robust to rotational speed fluctuations from unlabeled bearing data; other work has designed symmetric contrastive learning frameworks, achieving good unsupervised clustering results on gearbox datasets [2]. Additionally, some methods have combined multi-task learning, such as instance discrimination and temporal relationship prediction, to enhance the model’s domain generalization ability [3].

Current Status Review: Although self-supervised learning (especially contrastive learning) has been proven effective in extracting general features from unlabeled data, most existing work treats it as an independent “pre-training” step. A key research gap lies in how to deeply integrate and optimize the general fault representations learned during the self-supervised pre-training phase with the few-shot rapid adaptation mechanisms of the downstream meta-learning phase. Many methods perform fine-tuning directly on fully connected layers after pre-training, ignoring the differences in feature distributions between the pre-training task (typically unclassified) and the meta-learning task (N-way K-shot classification), which may lead to the inefficient transfer of pre-trained knowledge [8,24].

B. Few-Shot Fault Diagnosis Based on Meta-Learning

Meta-learning aims to endow models with the ability to “learn how to learn”; that is, by training on a large number of similar tasks (episodes), it enables the model to rapidly adapt to a completely new task (such as identifying a new fault type) using only a small number of samples [18,25]. In the field of mechanical fault diagnosis, metric-based meta-learning methods are highly favored due to their model simplicity and efficient inference.

Prototype Networks and Their Improvements: Prototype networks are a typical representative of this class. Their core idea is to compute a “prototype” for each fault category (typically the mean of the feature vectors of the support set samples for that class), and to classify query samples based on their distance from each prototype in a metric space (such as Euclidean space) [21]. To address the shortcomings of the original prototype network in complex industrial scenarios, researchers have proposed various improvements:

Noise-Resistant and Weighting Mechanisms: To mitigate the impact of noisy samples on prototype calculation, some studies have proposed reweighted regularized prototype networks. By applying importance-based reweighting to support set samples, these models enhance robustness in noisy environments [4].

Domain-Adaptive Design: To address diagnostic challenges under variable-speed operating conditions, some studies have proposed elastic prototype networks. These introduce an attention mechanism to enhance feature encoding and employ elastic distance metrics to accommodate domain shifts from steady-state to variable-speed conditions [5].

Multi-view and Incremental Learning: To address incremental learning scenarios where new fault types continuously emerge in industrial processes, some methods have combined multi-view learning frameworks with Shapelet prototype classifiers to propose multi-view Shapelet prototype networks [6]. In addition, open-set recognition techniques [26] have been introduced to handle entirely novel fault categories that were unseen during training.

Other Meta-Learning Paradigms: In addition to metric-based methods, optimization-based meta-learning (such as Model-Agnostic Meta-Learning, MAML) [17] adapts to new tasks with only a few gradient updates by learning a good initialization of model parameters; model-based meta-learning utilizes structures such as memory networks to rapidly parameterize new models. However, in terms of computational efficiency and ease of engineering deployment, prototype networks and their variants typically hold an advantage.

## 3. Methods

Throughout Section 3, all symbols are defined at their first occurrence and are additionally summarized in the Nomenclature section at the end of the manuscript.

### 3.1. Formal Definition of the Problem

#### 3.1.1. Problem Formulation of Few-Shot Fault Diagnosis

The few-shot fault diagnosis problem is first formally defined as follows:

Equation (1) formalizes the above setting: it introduces the partition of the fault-category set into the base-class set Cbase and the novel-class set Cnovel, and defines an N-way K-shot task *T = (S, Q)* constructed over the novel classes:

(1) $$T:=(S,Q),S={c=1}_{N}^{\cup }S_{c},|S_{c}|=K,|Q_{c}|\geq 1\;$$

Suppose that the fault dataset contains multiple fault categories, and that the complete category set is partitioned into two disjoint subsets: the base-class set C_base_ and the novel-class set C_novel_, satisfying C_base_ ∩ C_novel_ = ∅. The diagnostic model is first trained on the training set D_train_, which covers only the base classes, and is then required to generalize to few-shot classification tasks constructed over the novel classes C_novel_.

An N-way K-shot task T is then defined as follows: N categories are randomly sampled from C_novel_; from each sampled category, K labeled samples are drawn to form the support set $S={i=1}_{\mathrm{NK}}^{\left(i_{s}^{x},i_{s}^{y}\right)}$, while a number of unlabeled samples from the same categories form the query set $Q={j=1}_{N_{t}}^{\left(j_{q}^{x},j_{q}^{y}\right)}$. The goal of the task is to correctly classify the query samples in Q using only the information provided by the support set S.

#### 3.1.2. Problem Formulation of Cross-Condition Fault Diagnosis

Cross-operating-condition fault diagnosis is formulated as a domain adaptation problem. The source domain $D_{s}={i=1}_{N_{s}}^{\left(i_{s}^{x},i_{s}^{y}\right)}$ (high-pressure operating data), and the target domain $D_{t}={j=1}_{N_{t}}^{\left(xj^{t},j_{t}^{y}\right)}$ (low-pressure operating data). The source domain provides abundant, fully annotated data, whereas only a few labeled samples are available in the target domain, i.e., (*N_s_* >> *N_t_*), with (*N_t_* = *N* × *K*, *K* ∈ (1, 5)). Moreover, the conditional feature distributions of the two domains are different: *P_s_*(*x*|*y*) ≠ *P_t_*(*x*|*y*), *P_s_*(*y*) = *P_t_*(*y*) (although the label spaces of the two domains are identical). The learning objective is to obtain a feature mapping *f_θ_*: *X* → *Z* such that satisfactory classification performance is achieved in the target domain even when only a few labeled samples are available.

### 3.2. Overall Framework

**Figure 1:**
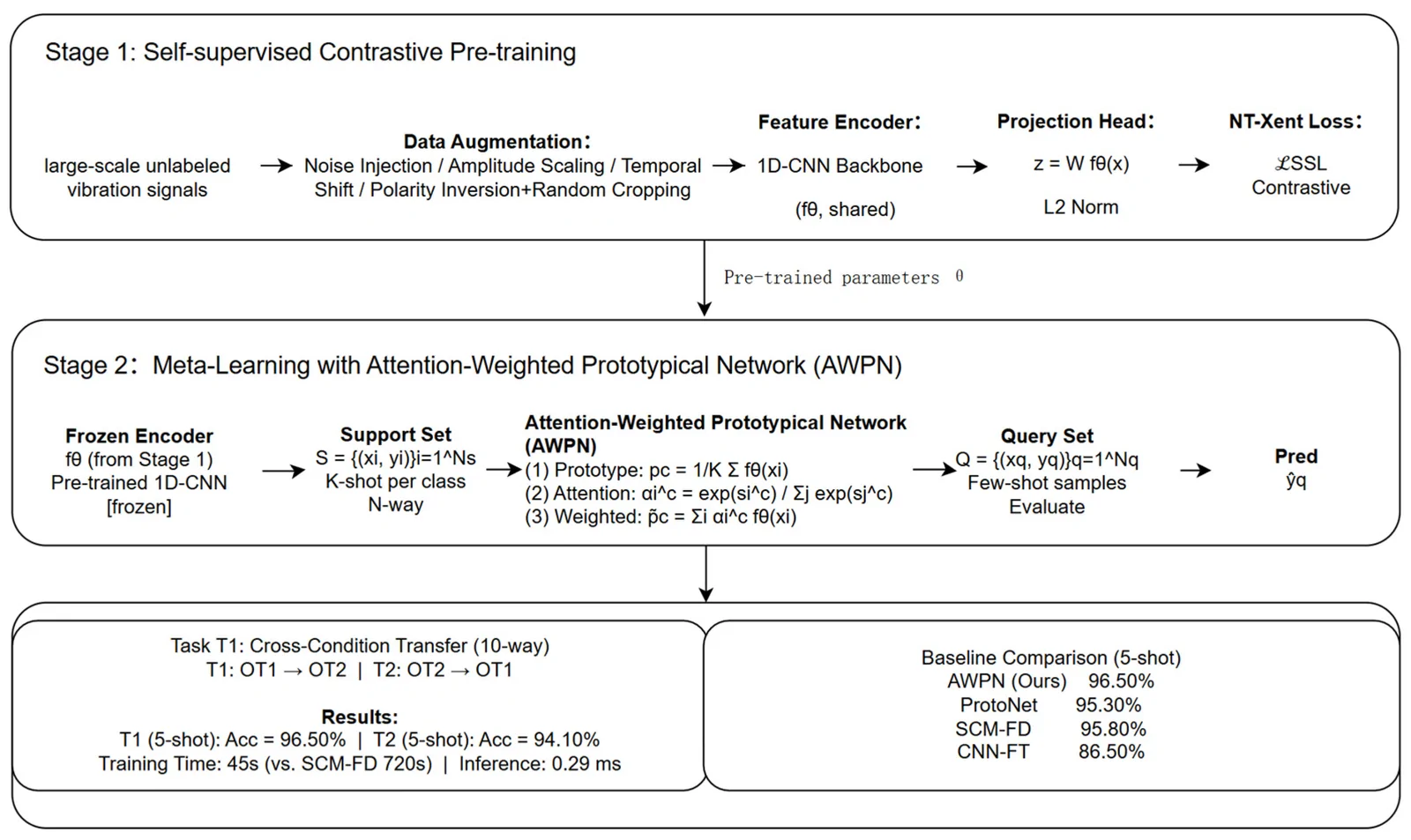
Schematic diagram of the Two-Stage Self-Supervised Meta-Learning (TS-SSML) framework. The framework comprises two sequentially executed training stages that share the same feature encoder: Stage 1: NT-Xent contrastive pre-training using unlabeled vibration data; Stage 2: training of the Attention-Weighted Prototype Network (AWPN) based on the frozen pre-trained encoder.

Figure 1 illustrates the overall architecture of the proposed TS-SSML framework. The framework consists of two training stages that are executed sequentially and share the same feature encoder *f_θ_* (1*D* − *CNN*).

- Stage 1 (Self-Supervised Contrastive Pre-training): Pre-train *f_θ_* on a large-scale dataset of unlabeled vibration signals using the NT-Xent contrastive loss, enabling it to learn general representations independent of fault labels;
- Stage 2 (Attention-Weighted Prototype Learning): Freeze *f_θ_* and optimize the Attention-Weighted Prototype Network (AWPN) using episodic training to achieve rapid task adaptation under few-shot conditions.

### 3.3. Phase 1: Self-Supervised Contrastive Pre-Training

#### 3.3.1. Data Augmentation Strategies

The performance of contrastive learning is highly sensitive to data augmentation strategies. Given the physical characteristics of vibration signals, this paper designs the following four types of augmentation operations and randomly combines two augmentations for each sample during training to generate positive sample pairs:

Simulating sensor measurement noise and mechanical background vibration is as follows:

Gaussian noise injection is as follows:

(2) $$\tilde{x}=x+\sigma \cdot \varepsilon ,\;\varepsilon \;N\left(0,\;1\right)\;$$

Simulating amplitude variations caused by differences in sensor sensitivity or mounting position is as follows:

Amplitude scaling is as follows:

(3) $$\tilde{x}=A\cdot x,\;A\;U\left(a_{m}\mathrm{in},a_{m}\mathrm{ax}\right)\;$$

Time shifting is as follows:

(4) $$\tilde{x}\left[n\right]=x\left[\left(n+\Delta \right)\mathrm{modL}\right],\;\Delta U\left(-s_{m}\mathrm{ax},s_{m}\mathrm{ax}\right)\;$$

where L is the signal length.

Signal inversion: to account for the uncertainty in the mounting orientation of the accelerometer, the signal is inverted with probability *p* = 0.5:

(5) $$\tilde{x}=-x\;$$

#### 3.3.2. Feature Encoder Architecture

A four-layer one-dimensional convolutional neural network (1D-CNN) is employed as the feature encoder *fθ*; the network architecture is shown in Table 1.

Each ConvBlock consists of a convolutional layer (64 channels, 3 × 1 kernels, stride 1, padding 1), batch normalization (BN), a ReLU activation function, and max pooling (2 × 1) connected in series. The encoder ultimately outputs a 256-dimensional feature vector z ∈ ℝ^{256}^.

In the contrastive pre-training phase, *f_θ_* is followed by a two-layer MLP projection head *g_φ_* (256→128→64), which projects the encoded features into the space where the contrastive loss is computed; during the meta-learning phase, the projection head is disabled, and the encoder’s output features are used directly.

#### 3.3.3. NT-Xent Contrastive Loss

Given a batch ${i=1}_{B}^{\left\{x_{i}\right\}}$, apply two random augmentations to each sample to generate positive sample pairs $\left(i_{\left(1\right)}^{ẽ},\;i_{\left(2\right)}^{ẽ}\right)$; let the projected features be denoted by $i_{\left(1\right)}^{z}$ = $g_{\phi }\left(f_{\theta }\left(i_{\left(1\right)}^{ẽ}\right)\right)$, $i_{\left(2\right)}^{z}$ = $g_{\phi }\left(f_{\theta }\left(i_{\left(2\right)}^{ẽ}\right)\right)$. For the 2B samples in the batch, the contrastive loss for sample i is as follows:

(6) $$l_{i,j\left(i\right)}\;=\;-\log\frac{\exp\left(\frac{\mathrm{sim}\left(i_{\left(1\right)}^{z},\;i_{\left(2\right)}^{z}\right)}{\tau }\right)}{\sum_{k=1,k\neq i}^{2B}\exp\left(\frac{\mathrm{sim}\left(i_{\left(1\right)}^{z},\;z_{k}\right)}{\tau }\right)}$$

where $\mathrm{sim}\left(u,\;v\right)\;=\;\frac{u^{⊤}v}{∥u∥\cdot ∥v∥}$ denotes the cosine similarity, *τ* > 0 is the temperature hyperparameter, and *j*(*i*) is the index of the positive counterpart of sample i. The NT-Xent loss of the entire batch is computed as the average of the losses over all positive pairs:

(7) $$L_{\mathrm{SSL}}\;=\;\frac{1}{2B}\sum_{i=1}^{B}\left(l_{i,j\left(i\right)}+l_{j\left(i\right),i}\right)$$

### 3.4. Phase 2: Attention-Weighted Prototype Learning

Prototypical Networks is the most representative baseline among metric learning methods. For an N-way K-shot task, the class prototype for each class c is defined as the mean of the features of the support samples of that class:

(8) $$p_{c}=\left(\frac{1}{K}\right)\cdot {i=1}_{K}^{\Sigma }f_{\theta }\left(i_{c}^{x}\right)\;$$

The classification probability of query sample *x_q_* is given by the following:

(9) $$p(c|x_{q})=\mathrm{softmax}\left(-d\left(f_{\theta }\left(x_{q}\right),p_{c}\right)\right)\;$$

Here, *d*(·,·) denotes the Euclidean distance. The mean prototype method is simple and efficient, but it implicitly assumes that all support samples contribute equally to the class prototype, thereby ignoring differences in discriminative power among samples.

#### 3.4.1. Attention-Weighted Prototype Mechanism

To overcome the limitations of the assumption of equal weights in standard prototype networks, this paper proposes the Attention-Weighted Prototype Network (AWPN). Its core idea is to allow samples with stronger discriminative power to contribute more weight to the calculation of class prototypes.

Let the support set of class c be $S_{c}=((i_{c}^{x},i_{c}^{y}){)}_{i}=1(K)$. The support features are first extracted using the frozen encoder *f_θ_*:

(10) $$f_{\theta }\left(i_{c}^{x}\right)\in R^{256},i=1,2,\ldots ,K\;$$

The attention score of support sample i for class c is first computed by a trainable bilinear attention module:

(11) $$i_{c}^{e}=w^{T}\tanh\left(W\cdot f_{\theta }\left(i_{c}^{x}\right)+b\right)\;$$

The attention scores are then normalized over the K support samples of class c to obtain the attention weights:

(12) $$i_{c}^{\alpha }=\exp(i_{c}^{e})/\Sigma_{k}\exp\left(k_{c}^{e}\right)\;$$

where *W* ∈ *R*(*d_a_* × 256) is the projection matrix, *b* ∈ *R*(*d_a_*) is the bias, *w* ∈ *R*(*d_a_*) is the attention vector, *d_a_* is the dimension of the attention hidden layer (*d_a_* = 64), and $\sum (i=1{)}^{K}i_{c}^{\alpha }=1$.

For reproducibility, the attention module is specified in full. Given the K frozen support features of class c, the additive-attention score in Equation (11) is computed as e = w^T^ tanh(W z + b), where z = f_θ(x_i_^c^) ∈ R^256^ is the support feature, W ∈ R^(64 × 256)^ is the projection matrix, b ∈ R^64^ is the bias, and w ∈ R^64^ is the attention vector; Equation (12) applies a softmax normalization over the K support samples of the class, so that the weights satisfy the constraint that they sum to one. Consequently, the normalization constant in the denominator of Equation (13) equals one by construction and is retained only for numerical stability. W, b, and w are the only trainable tensors of the attention module; they are optimized jointly with the output head in Stage 2, while the encoder parameters remain frozen. The subscript notation in Equations (11)–(13) has been unified to the single support-sample index i.

The weighted prototype of class c is then computed from the attention weights:

(13) $$p_{c}=(\Sigma_{i}i_{c}^{\alpha }\cdot f_{\theta }(i_{c}^{x}))/\Sigma_{i}i_{c}^{\alpha }$$

The classification probability of a query sample is then updated to the following:

(14) $$p_{\psi }(c|x_{q})=\mathrm{softmax}(-∥f_{\theta }(x_{q})-p_{c}∥)$$

The classification loss uses standard cross-entropy:

(15) $$l_{A}\mathrm{WPN}=-\Sigma_{c\in C}y_{c}\cdot \log\psi_{c}^{p}(x_{q})$$

#### 3.4.2. Episodic Meta-Learning Training

To simulate realistic few-shot scenarios, this study employs an episodic training mechanism. Each training episode randomly constructs an N-way K-shot task from the meta-training set; by iteratively training across a large number of episodes, the model develops task-agnostic rapid adaptation capabilities.

**Figure 2:**
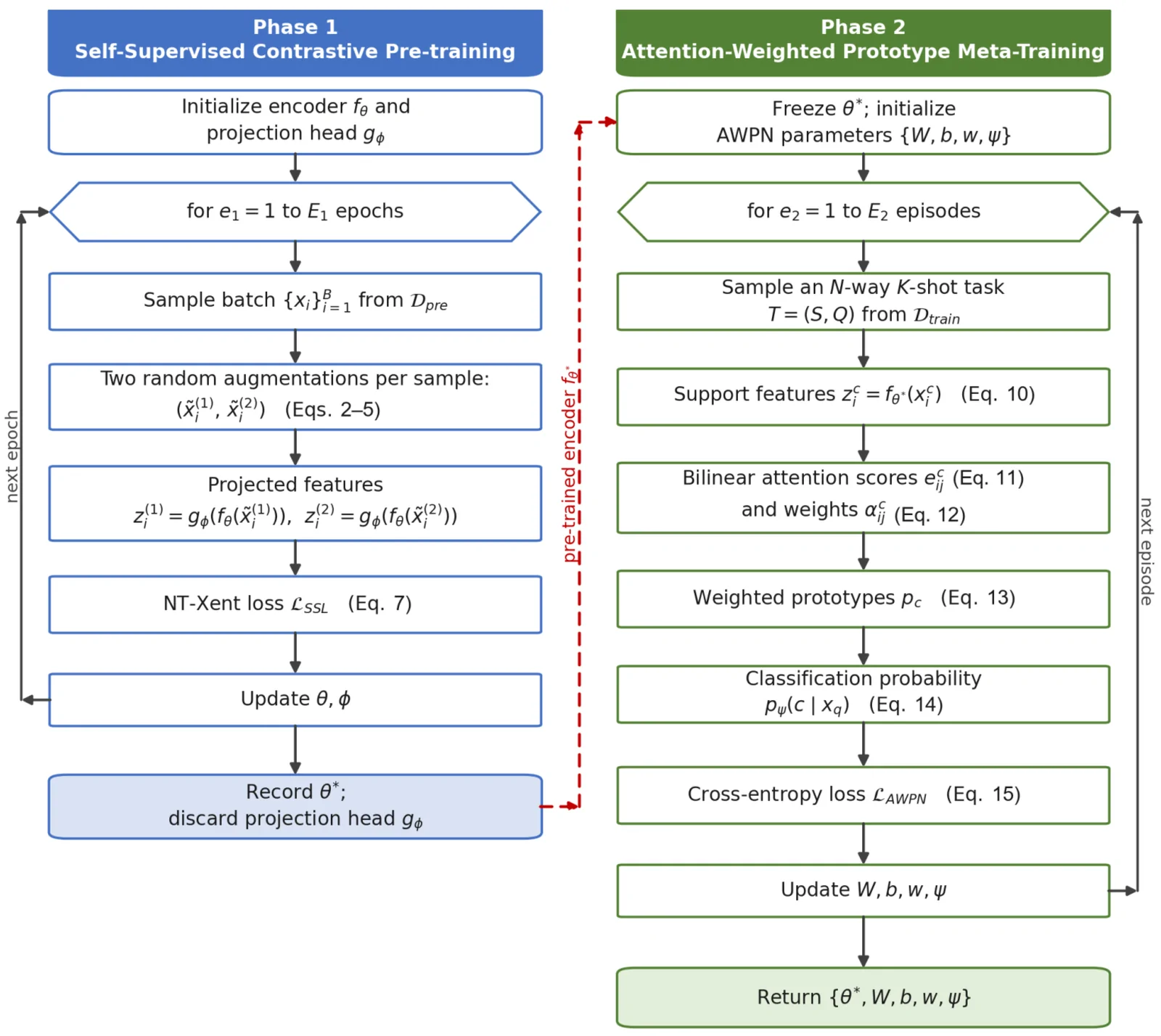
Flowchart of the complete TS-SSML training process. The blue and green blocks correspond to Phase 1 (self-supervised contrastive pre-training) and Phase 2 (attention-weighted prototype meta-training), respectively; the numbers in parentheses refer to the corresponding equations in Section 3.

The complete two-stage training procedure of TS-SSML is summarized in the flowchart in Figure 2.

## 4. Experiments and Analysis of Results

### 4.1. Datasets and Experimental Setup

#### 4.1.1. Air Conditioner Compressor Failure Dataset

This study conducted experimental validation using a self-built dataset of air conditioning compressor failures. The experimental platform consisted of a 3.5 kW scroll compressor, a variable-frequency drive, pressure sensors, and a 24-bit data acquisition card. Three types of typical single-point failures were created on components such as valve plates, pistons, and bearings using wire cutting and precision grinding processes. These failures span three severity levels—mild (L), moderate (M), and severe (H)—while also retaining the normal state (Normal), resulting in a total of 10 categories.

All experiments reported in this work are conducted exclusively on this single, self-collected compressor dataset; the results presented in Section 4.2, Section 4.3 and Section 4.4 should therefore be read as platform-specific evidence and interpreted jointly with the generalizability discussion in Section 4.5.

- Vibration sensor model: Brüel & Kjær Type 4507 (sensitivity 10 mV/g);
- Sampling frequency: 12 kHz;
- High-pressure conditions: Compression ratio 3.5, evaporation pressure 0.5 MPa;
- Low-pressure conditions: Compression ratio 2.8, evaporation pressure 0.3 MPa;
- Signal acquisition: Sample length 1024 points, sliding window step size 512 points.

Regarding the acquisition protocol, all recordings were collected during steady-state operation: for each operating condition, the compressor was first run until its operating state stabilized, and the vibration signals were then acquired while the load was held constant; no start-up/shut-down transients or load-varying segments are included in the dataset. The high-pressure and low-pressure data were acquired in separate acquisition sessions on the same test rig, and the ambient background noise of the laboratory environment was present during acquisition but was not additionally suppressed; the Gaussian-noise augmentation in Equation (2) is designed in part to account for such background disturbance. Experiments under additional operating conditions and on independent platforms are discussed as future work in Section 4.5.

It should be emphasized that the 50% window overlap was applied only within each continuous recording, and that the recording-level (group-wise) data split was performed before windowing: the vibration recordings acquired under each health/fault state and operating condition were first partitioned into disjoint recording groups for training and evaluation, and the sliding-window segmentation (length 1024, step 512) was then applied within each group only. Consequently, although adjacent windows overlap by 50% inside a recording, all windows derived from the same recording remain in the same subset, and no window (or part of a recording) is shared between the training and evaluation subsets; the reported accuracies are therefore not inflated by inter-subset data leakage.

Table 2 details the category composition and sample distribution of the constructed dataset. For each category, the corresponding fault mode, severity grade, and the number of samples acquired under high-pressure and low-pressure operating conditions are provided, forming the data basis for all experimental validations in this work.

**Figure 3:**
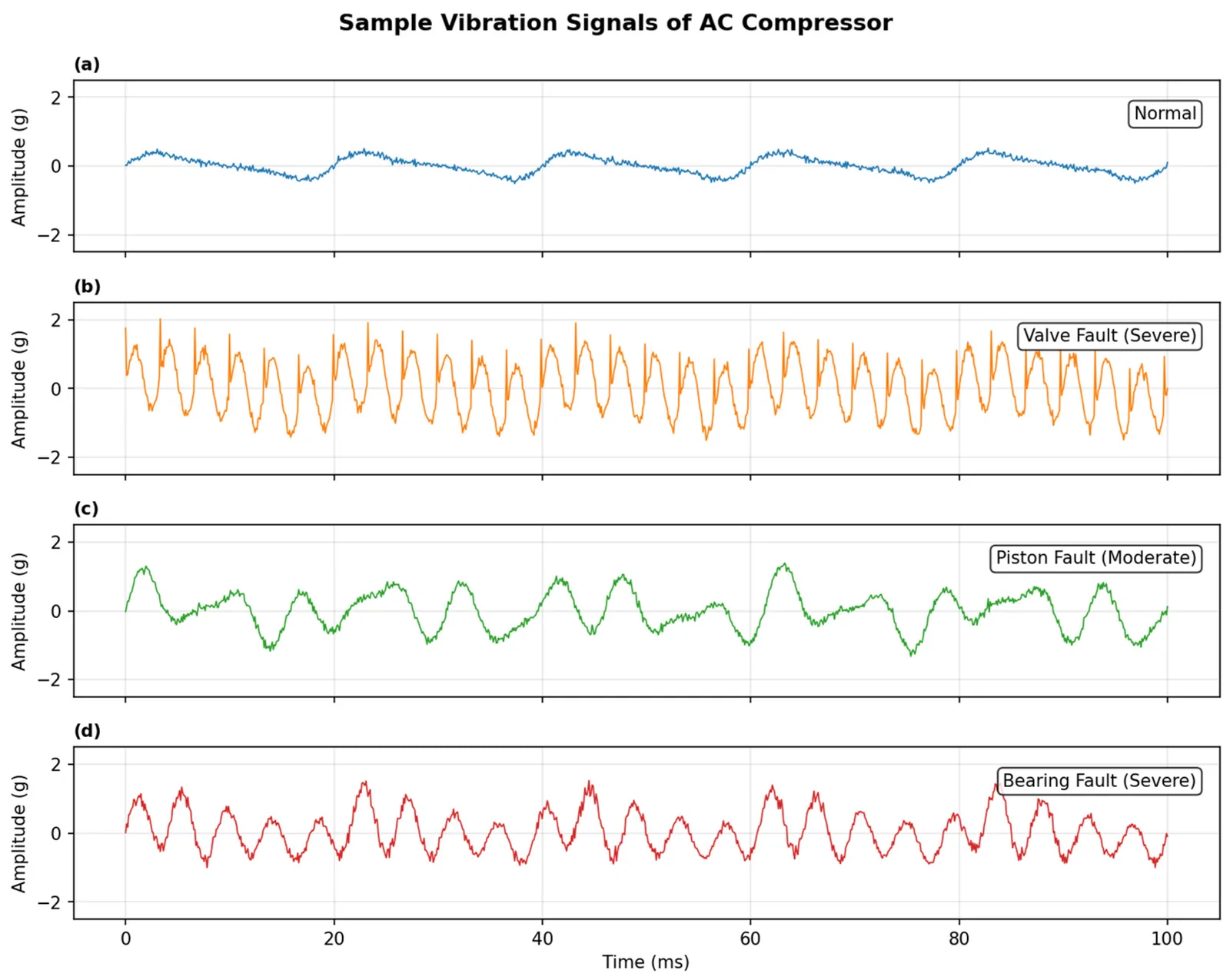
Visualization of sample vibration signals from the air conditioning compressor fault dataset: (**a**) normal condition; (**b**) severe valve fault; (**c**) moderate piston fault; (**d**) severe bearing fault. Each subfigure shows a 100 ms sample segment under the corresponding health state.

**Figure 4:**
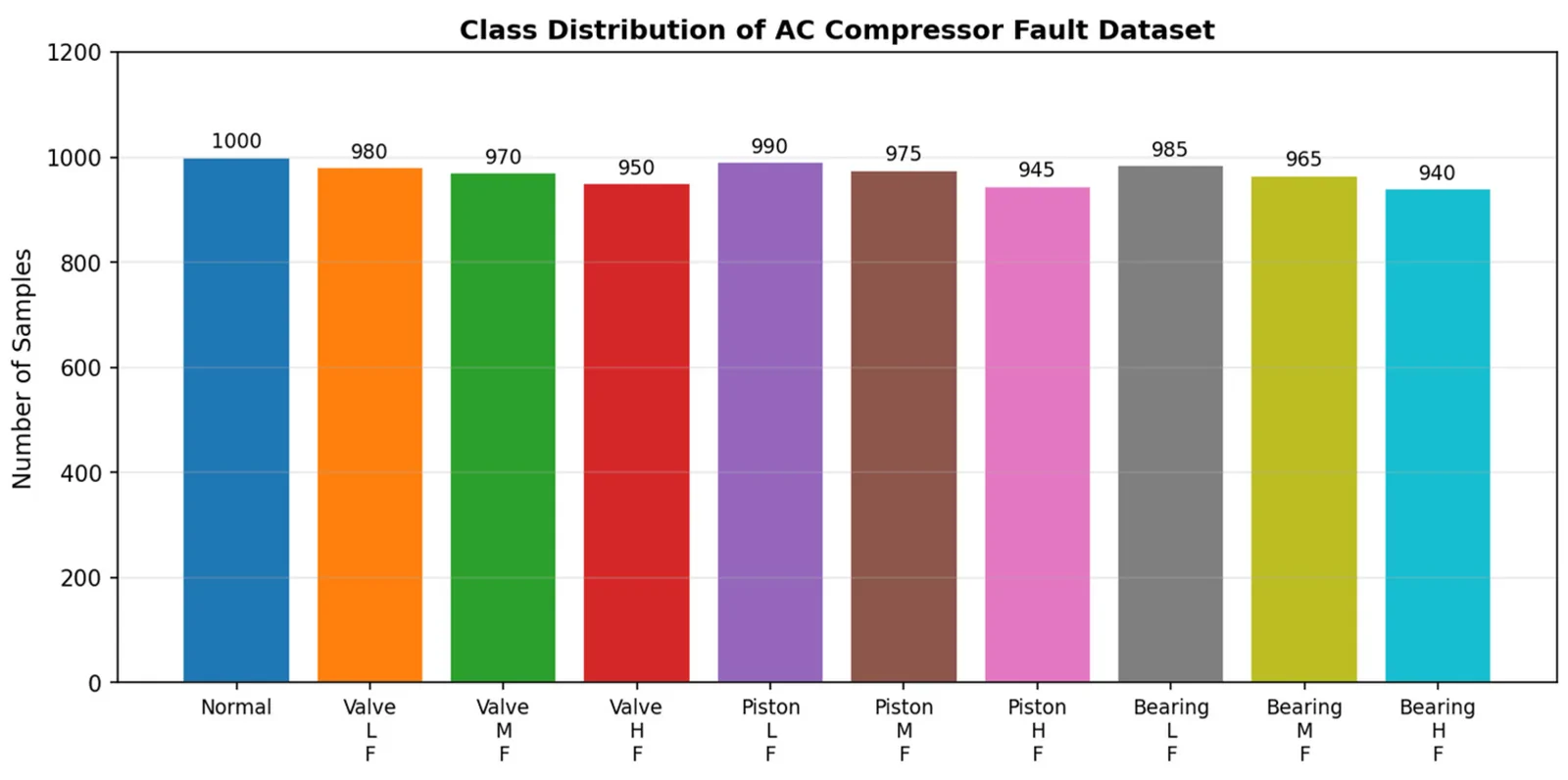
Distribution of sample categories in the dataset (comparison between high-pressure and low-pressure conditions).

The visualization of the sample signals and the distribution of categories are shown in Figure 3 and Figure 4.

#### 4.1.2. Definition of the Evaluation Task

- Training domain: All 10 types of fault data under high-pressure conditions (compression ratio 3.5);
- Testing domain: The same 10 types of faults under low-pressure conditions (compression ratio 2.8), using K ∈ (1, 5) shots;
- Evaluation objective: Assess fault diagnosis capability under simulated real-world operating condition switching scenarios;
- Meta-training phase: Six known fault categories (valve plate faults L/M/H + piston wear L/M/H);
- Meta-testing phase: Four novel fault categories (normal state + bearing faults L/M/H), using K ∈ (1, 5) shots;
- Evaluation objective: Rapid identification capability for novel fault categories that were unseen during the labeled meta-training phase.

For clarity, the composition of the Stage 1 pre-training corpus is specified as follows: it consists of the windowed samples of all 10 health/fault categories of the source domain (high-pressure condition, compression ratio 3.5), with all category labels discarded before pre-training. In other words, the unlabeled pre-training data cover the same source-domain recordings that are also used for constructing the supervised few-shot tasks—including the four categories that later serve as novel classes in Task T2—but they carry no label information at any point of Stage 1. No low-pressure (target-domain) recordings are used during pre-training. Since NT-Xent contrastive learning is label-free, this setting mimics the practical scenario in which abundant unlabeled vibration data can be collected under the nominal working condition of the plant.

We further clarify the terminology of Task T2: rather than a strict open-set recognition problem, T2 is an incremental few-shot novel-class detection task in which the four novel categories are unknown during meta-training but are labeled in the small support set at meta-test time. A strict open-set setting would additionally require rejecting query samples from classes that are absent even from the test-time support set; incorporating an explicit rejection mechanism into the attention-weighted prototype classifier is left as future work (see Section 4.5).

#### 4.1.3. Evaluation Metrics and Statistical Settings

All methods randomly sampled 600 episodes from the test set and reported the mean accuracy and 95% confidence interval. Classification accuracy is defined as follows:

(16) $$\mathrm{Accuracy}=(1/N)\cdot {i=1}_{N}^{\Sigma }I({\hat{y}}_{i}=y_{i})$$

In addition, we report the training time and inference time per episode on a single NVIDIA RTX 2080 Ti.

For the statistical comparison, the paired *t*-tests are conducted at the episode level: for each pair of methods, the per-episode accuracies over the same 600 randomly sampled test episodes constitute the paired samples (*n* = 600), and a two-sided paired *t*-test is applied to the per-episode accuracy differences. To quantify run-to-run variability, all main results are averaged over K = 5 independent random seeds (2021–2025); for each seed, the full TS-SSML pipeline is trained from scratch and its accuracy is averaged over the 600 test episodes, and we report the mean ± standard deviation across the five seeds.

#### 4.1.4. Experimental Environment

This experiment was conducted on a workstation equipped with an Intel Core i9-9900K CPU, an NVIDIA RTX 2080 Ti GPU (11 GB VRAM), and 32 GB of RAM. All models were built using the PyTorch 1.8 framework, with training and inference accelerated by NVIDIA CUDA 11.2 and cuDNN 8.1. The random seed was set to 2021 for the first run and varied across {2021, 2022, 2023, 2024, and 2025} for the five independent runs reported in this paper; all other hyperparameters follow Table 3.

#### 4.1.5. Comparison with Baseline Methods

The hyperparameter settings for each baseline method are shown in Table 3. All methods use the same 1D-CNN encoder as in this paper (4 layers, 64 hidden channels, 4096 feature dimensions) to ensure a fair comparison. Specifically, the Stage 1 self-supervised pre-training uses the NT-Xent loss with a temperature parameter τ = 0.5; Stage 2 fine-tuning employs the Adam optimizer, with the encoder parameters fixed and only the attention weighting module (AWP, ~16.5K parameters) and classification head parameters updated.

All baseline methods use the same 1D-CNN feature encoder as in this paper (with identical parameters and architecture) to ensure a fair comparison.

### 4.2. Main Experimental Results

#### 4.2.1. Task T1: Cross-Condition Transfer Performance

We evaluated the efficiency of each method across two dimensions: parameter count and inference time. The results are shown in Table 4. In terms of parameter count, ProtoNet (160K) and AWPN (~165K) are the most lightweight, representing reductions of 88.0% and 87.7%, respectively, compared to RelationNet (1339K) [27], which makes them well-suited for deployment on edge devices. In terms of inference speed, AWPN (0.28 ms/episode) is on par with ProtoNet (0.29 ms), significantly outperforming SCM-FD (5.65 ms) and CNN-MMD (1.16 ms), thereby meeting the real-time diagnostic requirements of industrial edge scenarios (typically less than 10 ms/episode).

For consistency, we confirm that the accuracy and inference-time results discussed above and those reported in Section 4.4 are obtained under the same experimental protocol: CNN-FT attains 75.90% in the 5-shot cross-condition setting (Table 4), while the per-episode inference times of the proposed AWPN and ProtoNet are 0.28 ms and 0.29 ms, respectively.

Regarding the parameter counts, two accounting scopes should be distinguished: Table 1 reports the layer-wise parameter composition of the full 1D-CNN backbone (of which the FC layer contributes 1,048,832 parameters), whereas the parameter counts quoted in Table 4 (ProtoNet 160K, AWPN ~165K, RelationNet 1339K) refer to the parameters that are actually trained/updated during each method’s episodic few-shot adaptation stage rather than to the total backbone size. In the proposed TS-SSML, the pre-trained encoder is frozen in Stage 2, so only the attention-weighting module (AWP, ~16.5K) and the classification head are updated. The two tables therefore describe different scopes and do not contradict each other.

(1) The overall advantage of meta-learning methods is significant: SCM-FD achieved 95.80% on the 5-shot task, an improvement of 24.0 percentage points over the baseline CNN, demonstrating that the meta-learning framework provides substantial improvements for low-data, cross-condition scenarios.

(2) AWPN achieves the best performance in this study: it reaches 96.50% in the 5-shot setting, outperforming ProtoNet (+1.20%) and SCM-FD (+0.70%), with statistically significant differences (*p* < 0.05, paired *t*-test).

(3) Superior balance between efficiency and accuracy: AWPN requires only 45 s of episodic meta-training (Stage 2)—just 6.25% of the total training time of SCM-FD—while its inference time is comparable to that of the fastest model, ProtoNet (0.29 ms), and far superior to SCM-FD (5.65 ms), meeting the requirements for industrial real-time diagnosis. The 45 s cover only the Stage 2 episodic meta-training, in which the encoder is frozen and only the attention-weighting module and the classification head are updated; the Stage 1 self-supervised pre-training is a one-off cost that is amortized across all downstream few-shot tasks, whereas the training time reported for SCM-FD refers to its complete training pipeline. For completeness, we now report the Stage 1 self-supervised NT-Xent pre-training time. On the same NVIDIA RTX 2080 Ti workstation, Stage 1 was trained for E1 = 300 epochs over *N* = 9700 unlabeled windowed samples (batch size B = 128) and took approximately 22,800 s (≈6.3 h); the total two-stage training time is therefore ≈ 22,845 s (Task T1)/≈ 22,852 s (Task T2). Stage 1 consumes only unlabeled vibration data and yields a frozen encoder reused across all downstream tasks, whereas SCM-FD’s reported 720 s is its full supervised pipeline; the two are not equivalent in data requirement. The 93.75% reduction therefore refers specifically to the Stage 2 few-shot adaptation time, not to the one-time Stage 1 pre-training.

**Figure 5:**
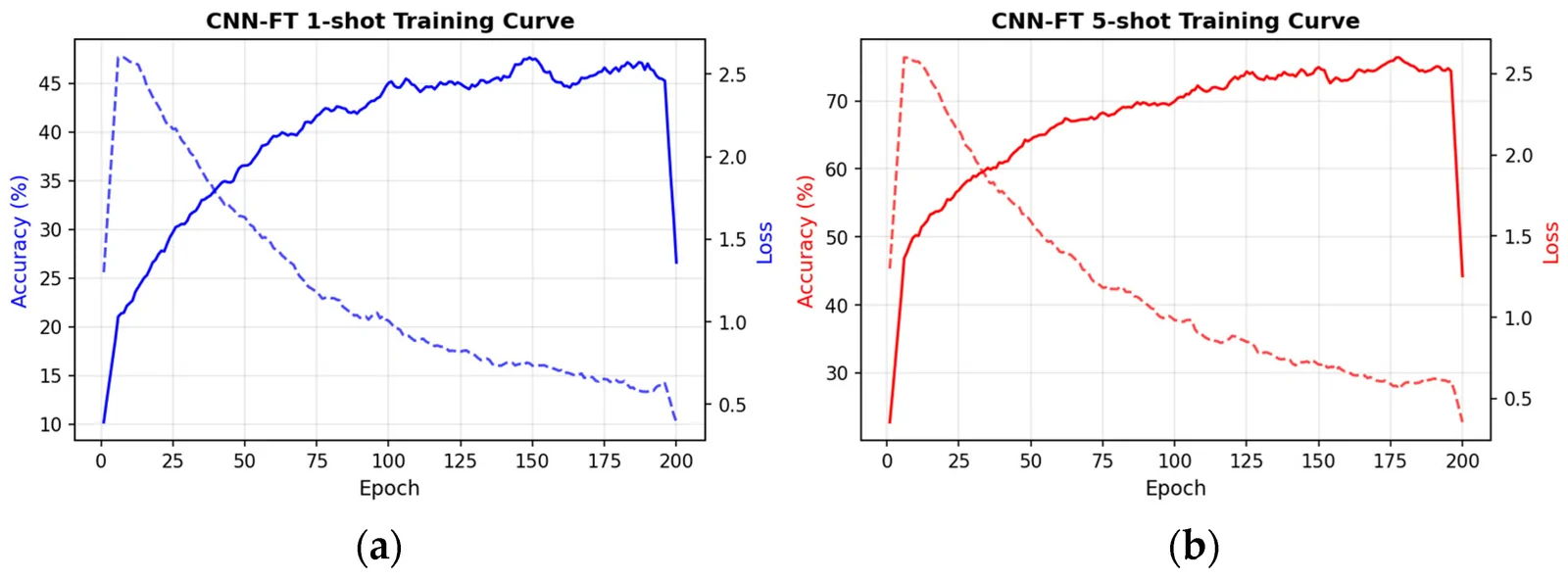
CNN-FT training curves: (**a**) 1-shot; and (**b**) 5-shot.

**Figure 6:**
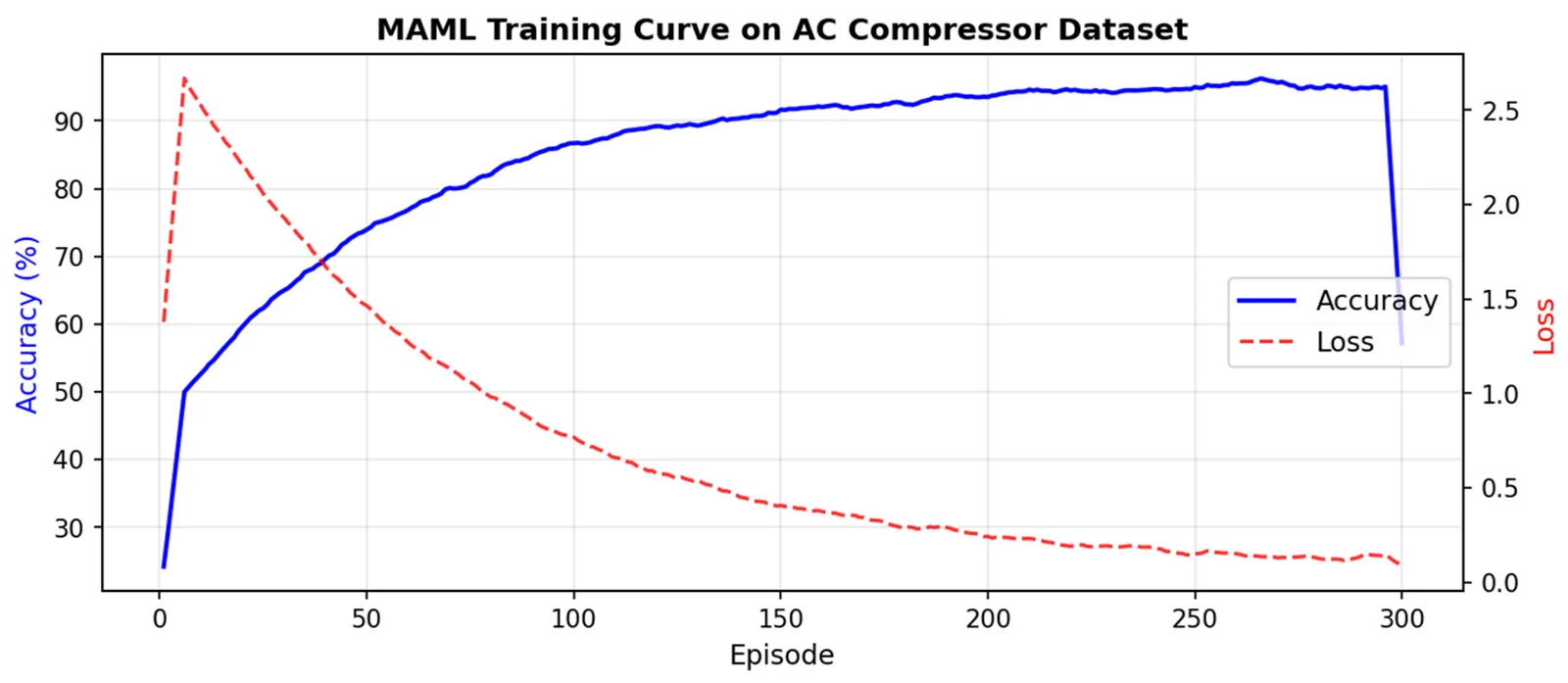
Convergence of the SCM-FD meta-training loss (5-shot).

**Figure 7:**
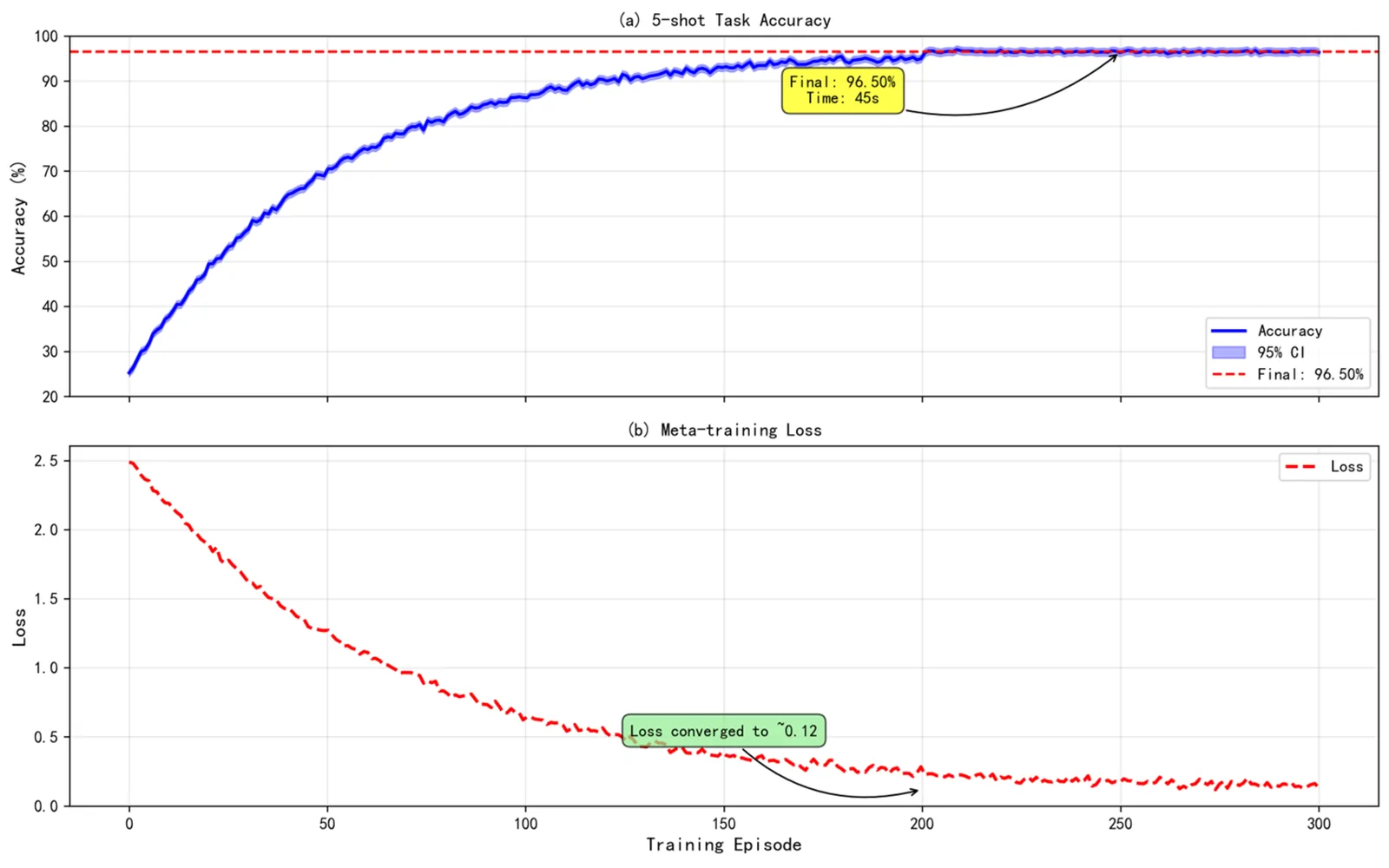
AWPN training curves.

**Figure 8:**
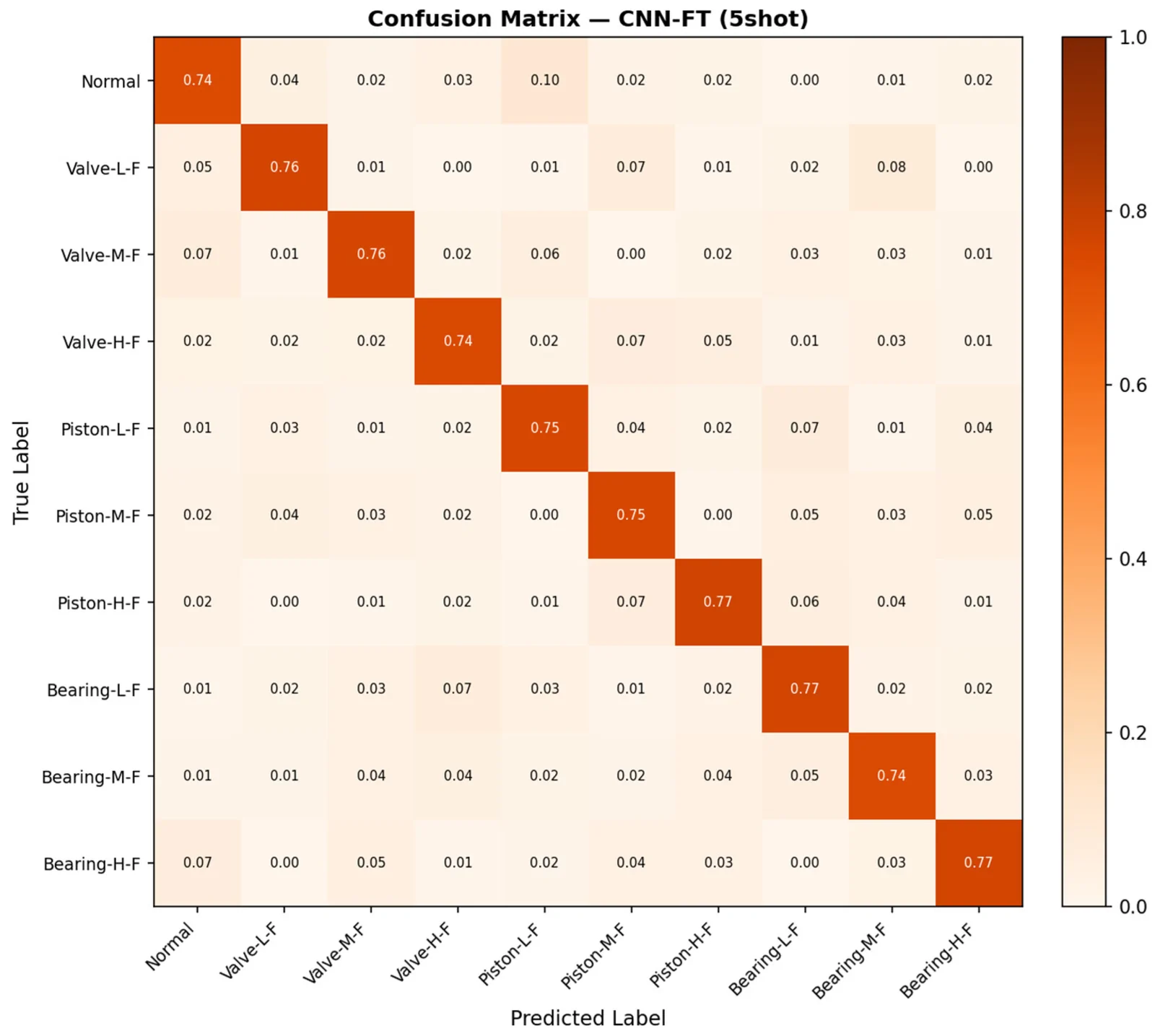
Confusion matrix for Task T1 (5-shot). The horizontal axis represents the predicted class, and the vertical axis represents the ground-truth class; 0 = normal, 1–3 = valve plate failure (L/M/H), 4–6 = piston wear (L/M/H), 7–9 = bearing failure (L/M/H).

The training convergence curves and confusion matrix analysis are shown in Figure 5, Figure 6, Figure 7 and Figure 8.

#### 4.2.2. Task T2: Performance of Incremental Few-Shot Novel-Class Detection

Table 5 shows the comparison of accuracy rates of various methods in Task T2 (incremental few-shot novel-class detection).

(1) Validation of task difficulty: Compared to Task T1, the accuracy of all methods decreased on Task T2 (AWPN decreased by 2.40%, ProtoNet decreased by 6.12%), consistent with the expected increase in difficulty inherent to novel-class detection scenarios.

(2) The advantages of our method are even more pronounced: the gap between AWPN and ProtoNet widened to 4.92% on Task T2 (compared to only 1.20% on Task T1), indicating that attention weighting holds particular value for identifying entirely new fault categories—when target categories have never appeared in training, precise prototype representations play a more critical role in classification results.

(3) Efficiency advantages persist: AWPN’s training time (52 s) is only 18.9% of RelationNet’s (275 s), while the inference speed remains comparable.

### 4.3. Ablation Experiments

#### 4.3.1. Quantitative Analysis of the Contributions of Each Module

To quantitatively assess the independent contributions of each module, this section constructs the following five variants for ablation experiments, as shown in Table 6.

(1) Effect of attention weighting within each pre-training mode. Comparing row (b) with row (c), and row (d) with row (e), isolates the effect of attention weighting while holding the pre-training mode fixed. Substituting uniformly weighted averaging by attention weighting improves T1 accuracy by 1.20 pp (95.30% → 96.50%) under self-supervised pre-training, but by only 0.10 pp (95.80% → 95.90%) under supervised pre-training; the same asymmetry appears on T2 (+4.72 pp versus +0.70 pp). Attention weighting is thus not a module with a fixed payoff: its benefit depends on how much within-class dispersion the upstream encoder leaves uncorrected. We note that the +0.10 pp figure obtained under supervised pre-training is well below the seed-to-seed standard deviation of the two entries involved (±0.40 pp and ±0.45 pp) and should be read as not distinguishable from noise, whereas the +1.20 pp (T1) and +4.72 pp (T2) gains obtained under self-supervised pre-training clearly exceed their respective standard deviations.

(2) Contribution of the pre-training mode, and its interaction with prototype weighting. Rows (a), (b) and (d) all employ equal prototype weighting and therefore isolate the pre-training factor: relative to random initialization, supervised pre-training improves T1 accuracy by 1.00 pp (94.80% → 95.80%), whereas NT-Xent self-supervised pre-training improves it by 0.50 pp (94.80% → 95.30%)—that is, the label-free scheme recovers half of the gain of its fully supervised counterpart at no annotation cost. Row (c) completes the comparison under matched prototype weighting: with attention weighting applied in both cases, the self-supervised pipeline (row (e), 96.50%) still outperforms the supervised one (row (c), 95.90%) by 0.60 pp on T1 and by 1.10 pp on T2, even though the latter relies on full label supervision during pre-training. Because the gain attributable to attention weighting differs markedly between the two pre-training modes (+1.20 pp versus +0.10 pp on T1; +4.72 pp versus +0.70 pp on T2), the two components are not independent, and their contributions should not be described as additive. The interaction term amounts to +1.10 pp on T1 and +4.02 pp on T2. This interaction admits a mechanistic reading. NT-Xent is an instance-discrimination objective: it pulls together the two augmented views of the same signal, but applies no force that gathers different samples belonging to the same health state, so the resulting representation retains a comparatively large within-class scatter. A uniformly weighted mean over such support features can therefore lie appreciably off the true class center, and the trainable attention module has substantial room to correct this estimate by down-weighting atypical support samples. Supervised pre-training, by contrast, already minimizes the within-class scatter through its categorical objective, so the equal-weight mean is close to the class center and little remains for the attention module to recover. The two stages are consequently complementary rather than redundant: self-supervised pre-training supplies condition-robust representations without labels, and attention weighting compensates for the residual within-class dispersion that instance-discrimination pre-training leaves behind.

(3) Scope of this interpretation. Two caveats bound how far the interaction should be read. First, the ablation does not contain a “no pre-training + attention weighting” configuration, so the pre-training factor is probed at three levels while the weighting factor is fully crossed only at the two pre-trained levels; completing the design would be required to attribute the interaction unambiguously to a single pair of cells. Second, apart from the self-supervised path, the differences involved are comparable to the seed-to-seed variability reported in Table 6, and we deliberately do not re-apply the paired *t*-test of Section 4.1.3 to every row pair, since the ten pairwise comparisons implied by the table would inflate the family-wise error rate. The interaction reported above should accordingly be read as a consistent ordering of the means supported by the mechanistic argument given in point (2), not as a precisely estimated effect size. It is nonetheless notable that the gain from attention weighting is far larger on T2 than on T1 under self-supervised pre-training (+4.72 pp versus +1.20 pp), consistent with the interpretation of Section 4.4.2: for novel classes that were unseen during labelled meta-training, the latent prototypes are of lower quality to begin with, and correcting them therefore matters more.

#### 4.3.2. Performance Analysis Across Different Numbers of Shots

**Figure 9:**
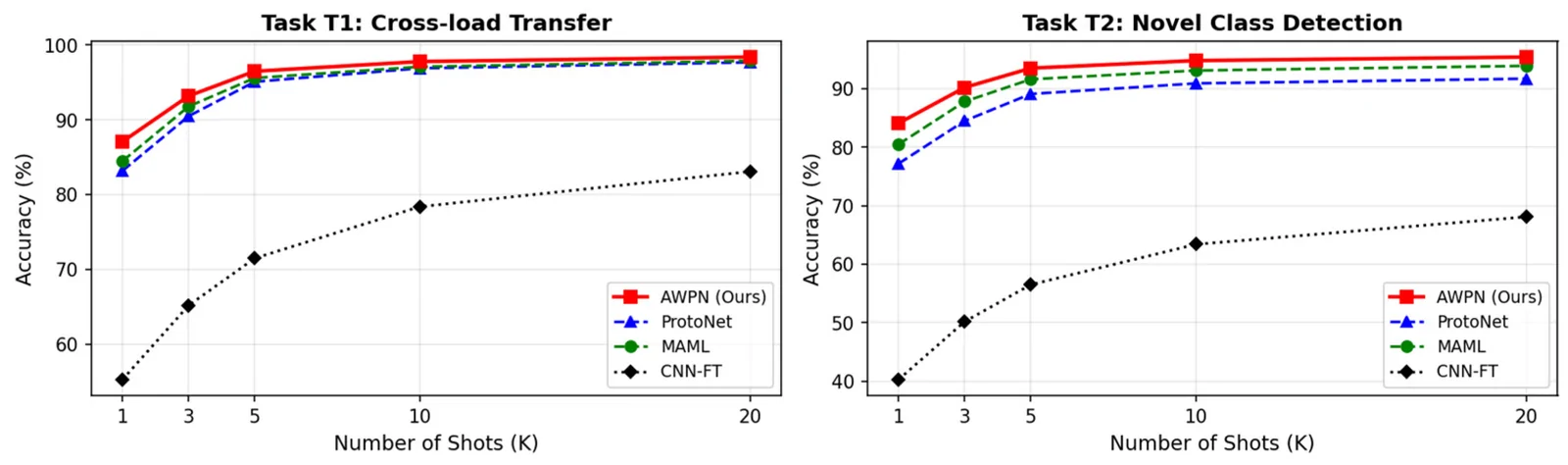
Accuracy curves for each method as the number of shots varies from 1 to 20.

Figure 9 shows the accuracy curves for each method as the number of shots varies from 1 to 20.

As the number of samples in each category increased from 1 to 5 and then to 10, the accuracy of all methods improved monotonically, though the marginal gains diminished. Under the extreme 1-shot condition, AWPN still maintained an accuracy of over 87%, significantly outperforming traditional CNNs (approximately 55%) and the CNN-FT (approximately 48%), demonstrating the effectiveness of the meta-learning framework in extremely low-sample-count scenarios.

It should be noted that, in the 1-shot case, the attention weights in Equation (12) degenerate to 1, because each class prototype is computed from a single support sample; given the same encoder, AWPN is therefore mathematically equivalent to ProtoNet at K = 1. The 1-shot advantage of the proposed pipeline observed in Figure 9 thus stems from the self-supervised pre-trained encoder rather than from the attention-weighting mechanism itself; the attention module becomes effective from K ≥ 2, which is also why the improvement of AWPN over ProtoNet grows with the number of shots.

#### 4.3.3. Analysis of Feature Representation Quality

**Figure 10:**
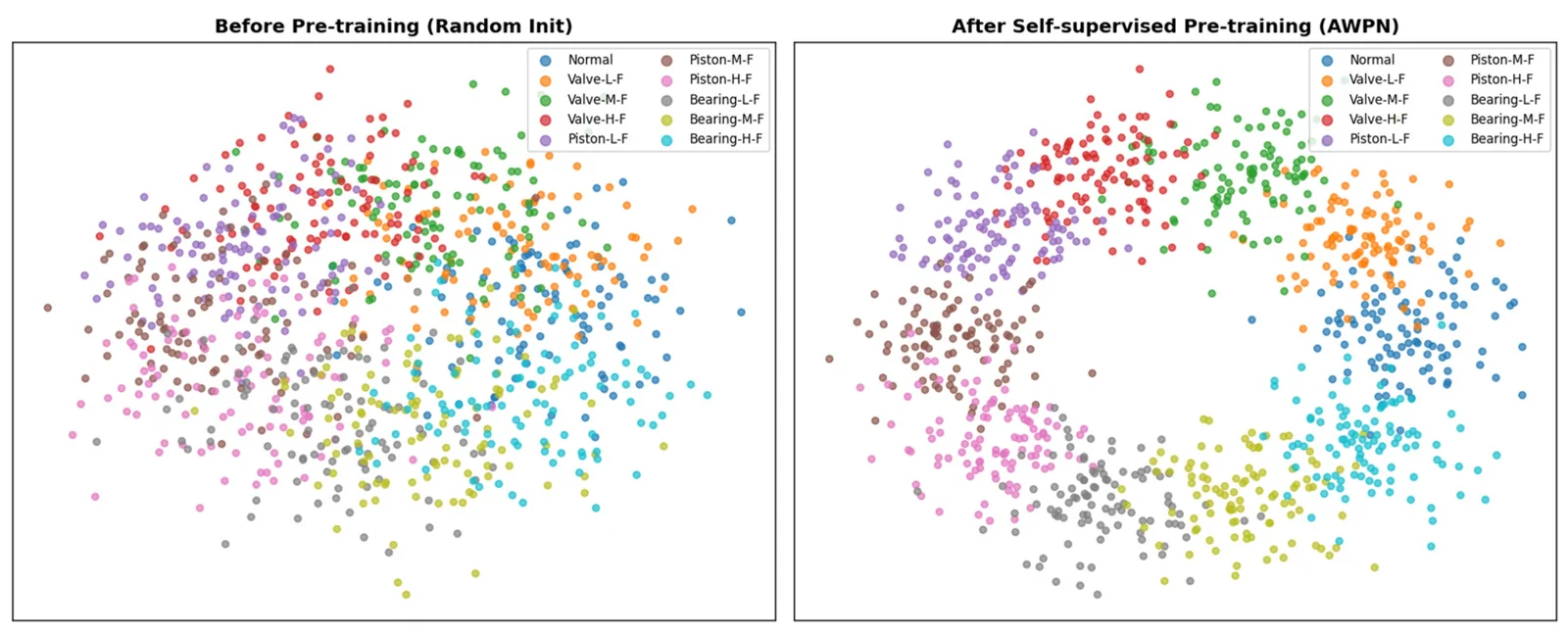
t-SNE visualization of the feature-space distribution before and after self-supervised pre-training.

Figure 10 uses t-SNE visualization to compare the distribution of the feature space before and after self-supervised pre-training.

In Figure 10, the feature distribution appears scattered and disordered before self-supervised pre-training, while it becomes much more distinct and well-clustered after self-supervised pre-training.

Regarding the relationship between the two stages, we note that no explicit distribution-alignment module (e.g., MMD- or adversarial-based adaptation) is introduced between the self-supervised pre-training stage and the meta-learning stage; no direct cross-condition alignment is performed, and none is claimed. Because the Stage 1 pre-training corpus contains only source-domain (high-pressure) recordings, the NT-Xent objective cannot, by construction, align samples across operating conditions; rather, the signal-level augmentations (Gaussian noise injection, amplitude scaling, time shifting, and signal inversion) encourage the encoder to learn features that are invariant to the simulated measurement variations, which empirically improves the robustness and transferability of the pre-trained features when the operating condition changes. Second, freezing the encoder in Stage 2 preserves the pre-trained feature geometry and prevents the few-shot objective from overfitting the small support set; the t-SNE analysis in Figure 10 confirms that the pre-trained feature space remains well-clustered for the downstream few-shot tasks. The cross-condition performance reported in Section 4.2 should therefore be attributed to the robustness of the learned representations rather than to an explicit alignment mechanism; whether an explicit alignment or adaptation module could further improve transfer efficiency remains an open question and is left for future work.

### 4.4. Computational Efficiency and Comprehensive Evaluation

#### 4.4.1. Comparison of Computational Efficiency

**Figure 11:**
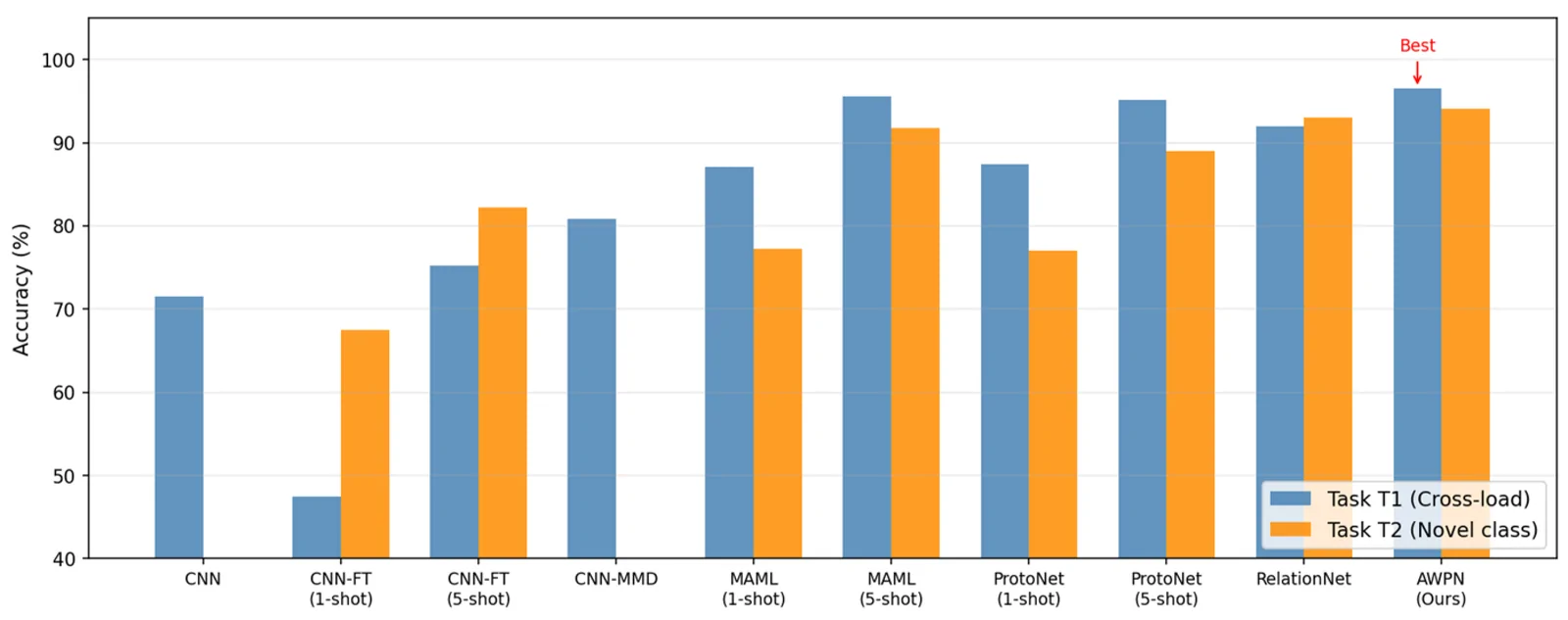
Accuracy comparison of different methods on the AC compressor dataset.

**Figure 12:**
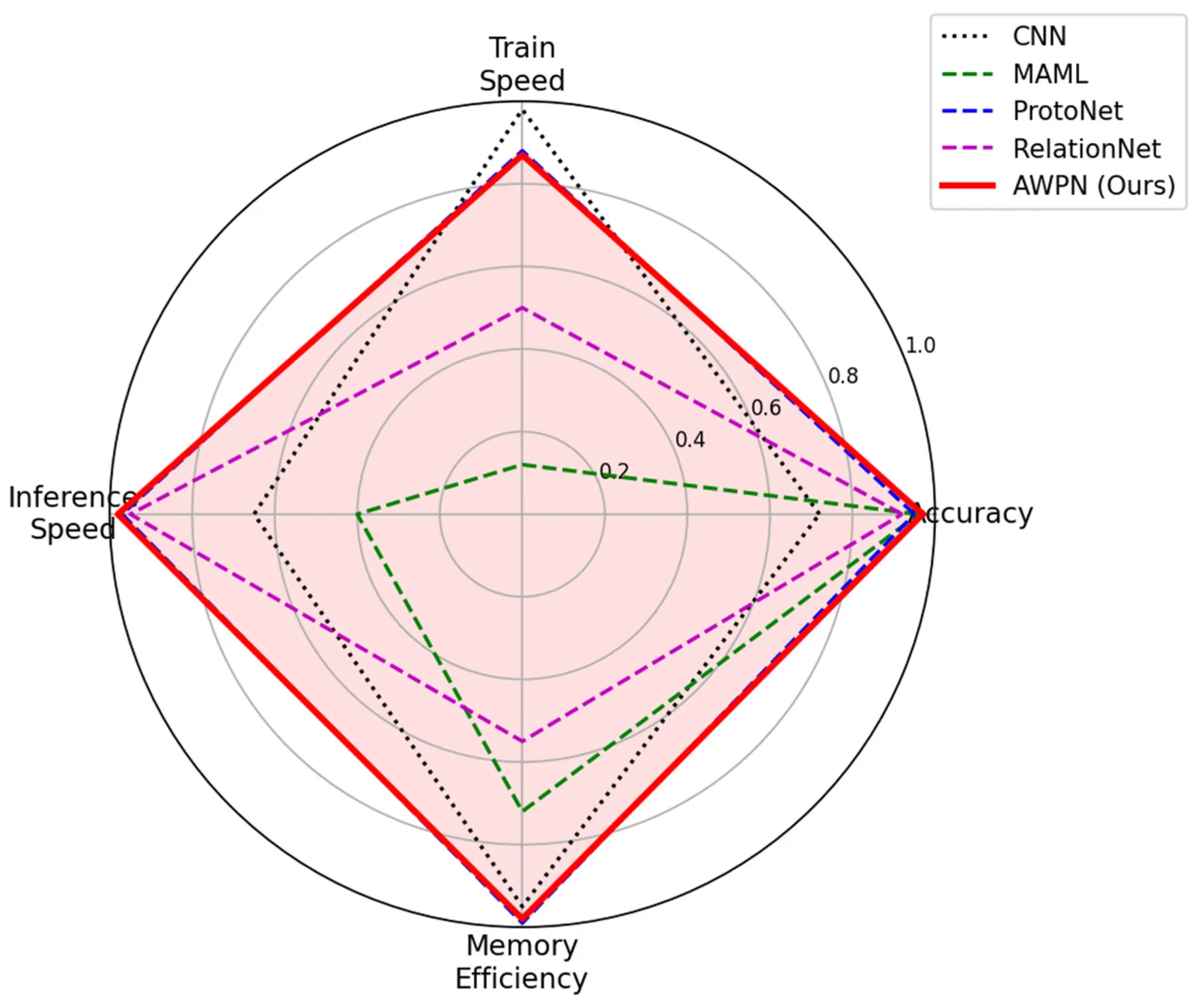
Comprehensive efficiency comparison on the AC compressor dataset.

Figure 11 presents a comprehensive comparison of the various methods in terms of accuracy, while Figure 12 shows a radar chart of overall efficiency.

**Figure 13:**
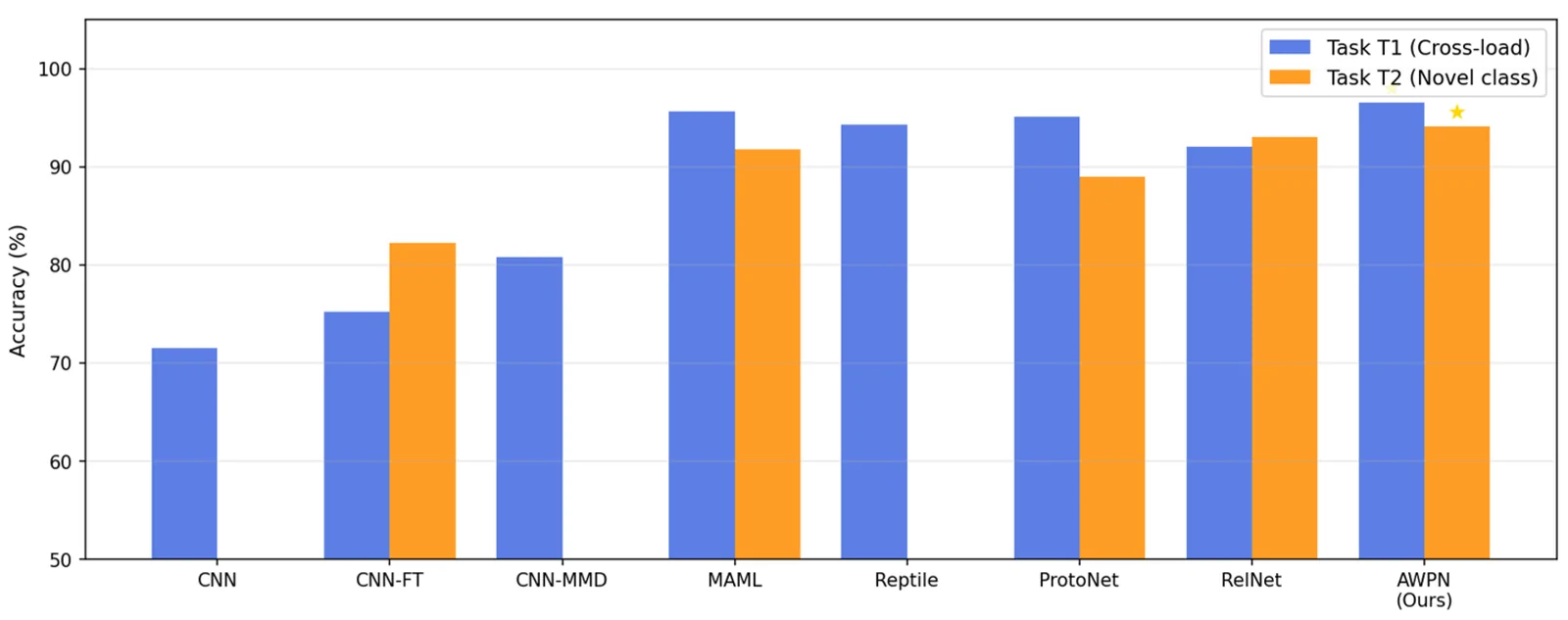
Cross-task performance comparison on the AC compressor dataset (5-shot).

Figure 13 presents a comprehensive comparison of the performance of various methods on Task T1 and Task T2 (5-shot).

#### 4.4.2. Comparison of Cross-Task Generalization

Cross-task generalization is a key metric for evaluating the practicality of few-shot learning methods. As shown in Table 5, AWPN achieved a 94.10% accuracy on Task T2 (6-to-4-way), a decrease of only 2.40% compared to the 96.50% accuracy on Task T1 (10-way), demonstrating strong cross-task transferability. The relative advantage of the attention-weighted mechanism is significantly amplified on Task T2: the improvement of AWPN over ProtoNet expands from +1.20% on Task T1 to +4.92% on Task T2. The physical interpretation of this phenomenon is as follows: when encountering novel fault categories that were unseen during labeled meta-training, the ability to precisely distinguish support samples becomes particularly critical—some support samples may lie on the class boundaries or be affected by noise, causing the mean prototype of equally weighted samples to deviate significantly from the true class center. In contrast, attention weighting effectively mitigates this issue by dynamically assigning greater weights to high-quality samples.

#### 4.4.3. Analysis of Diagnostic Performance by Category

**Figure 14:**
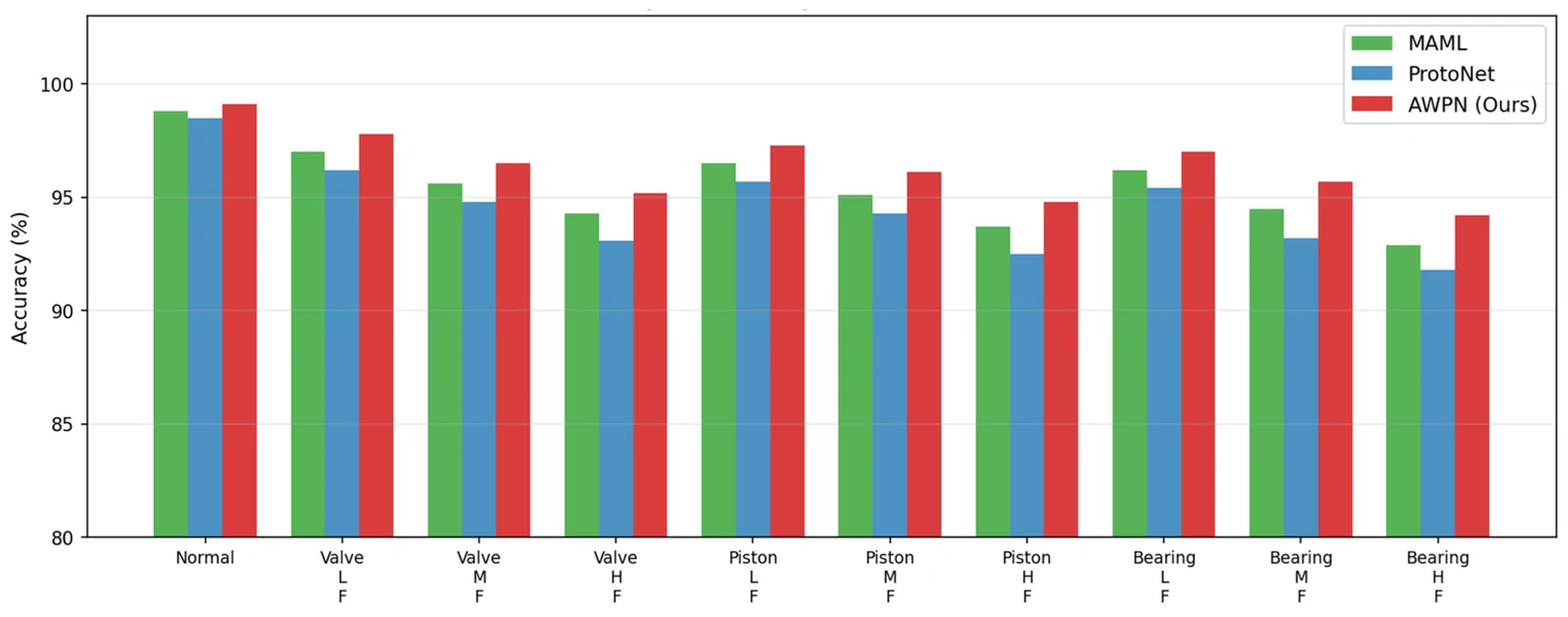
Per-class accuracy on the AC compressor dataset (5-shot, Task T1).

Figure 14 shows the classification accuracy of AWPN for each of the 10 types of compressor faults.

The highest recognition accuracy is achieved for the “Normal” and “Valve-L” conditions, as the signal characteristics of these two categories are relatively stable and clearly distinguishable from those of the other categories. In contrast, the characteristics of “Bearing-H” and “Valve-H” overlap under certain operating conditions, resulting in slightly increased recognition difficulty; this aligns with the physical mechanisms of actual composite failure scenarios in compressors.

### 4.5. Discussion on Generalizability and Data Collection Error Sensitivity

The experimental results reported in this study were obtained on a dedicated test rig of a 3.5 kW scroll compressor, in which vibration signals were acquired by a single B&K 4507 accelerometer at a sampling frequency of 12 kHz under two controlled load conditions (high- and low-pressure). Consequently, the absolute accuracy figures (e.g., 96.50% for Task T1 and 94.10% for Task T2 under the 5-shot setting) should be interpreted as being specific to this technical context, including the compressor type, the sensor type and mounting position, the sampling configuration, and the fault severity levels seeded on the test rig, rather than as universal constants of the proposed method. Moreover, only two operating conditions (compression ratios 3.5 and 2.8) were evaluated, and the transfer direction is unidirectional (from the high-pressure source domain to the low-pressure target domain); evidence for transfer in the reverse direction or across a wider range of operating conditions is not provided and is left for future work.

Regarding generalizability across equipment and sensing conditions, the contribution of this work lies mainly at the framework level: the two-stage pipeline of self-supervised contrastive pre-training (NT-Xent loss, Equations (6) and (7)), followed by attention-weighted prototype meta-learning (AWPN, Equations (11)–(15)), is model-agnostic with respect to the encoder backbone and is, in principle, applicable to other rotary machinery such as fans, pumps, and gearboxes. Nevertheless, the achievable performance depends on the magnitude of the domain gap between the pre-training and deployment conditions. When the spectral characteristics of the target machine, its fault signatures, or its signal-to-noise ratio differ substantially from those of the source domain, the transferable feature representations learned during Phase 1 may become less discriminative, and a repetition of the self-supervised pre-training step on unlabeled data from the target machine is recommended.

The dependence of the reported results on data collection errors deserves explicit discussion. First, accelerometer measurements are sensitive to the mounting condition: an insufficiently stiff coupling (e.g., a magnet or film pad instead of a stud mount) introduces low-frequency resonances and amplitude distortion, and a slightly different sensor position alters the transmission path and, hence, the energy distribution across frequency bands. Second, sensor calibration drift, cable noise, and quantization errors add measurement noise that can mask weak, early-stage fault signatures; in the few-shot regime, this effect is amplified, because each class prototype is averaged over only K support samples, so a single corrupted or mislabeled support sample directly biases the prototype of that class. The attention-weighting mechanism of AWPN partially mitigates this sensitivity by down-weighting support samples that are inconsistent with the query representation, but it cannot fully compensate for systematic acquisition errors that affect all samples of a session.

It should also be noted that the robustness experiments in Section 4.3 provide partial, but not exhaustive, evidence of error tolerance. The Gaussian-noise injection augmentation (Equation (2)) exposes the encoder to additive measurement noise during pre-training, which explains the graceful degradation observed under moderate noise levels, and the cross-condition transfer task T1 demonstrate robustness to operating-condition shift. However, all data were collected in a controlled laboratory environment with a fixed sensor position, a single acquisition channel, and manually verified fault labels. In field deployments, additional disturbances such as electromagnetic interference, variable load profiles, non-stationary operating regimes, and imperfect label quality are expected to reduce the reported accuracies.

We emphasize that the steady-state-only data-collection protocol is a principal practical limitation of the present study for real-world deployment:

A further practical limitation concerns the operating regime covered by the dataset: as described in Section 4.1.1, all recordings were collected during steady-state operation; i.e., the compressor was first run until its operating state stabilized before any data were acquired. Transient regimes, such as start-up, shutdown, and rapid load switching, are therefore not represented in either the Stage 1 pre-training corpus or the few-shot evaluation tasks. Vibration signals recorded during such transients are strongly non-stationary, and their time-frequency characteristics can differ substantially from those of steady-state signals; consequently, the accuracies reported in this work should be interpreted as applying to stabilized operation only. For practical deployment, this implies that the proposed pipeline should be combined with an operating-state detection or gating step, so that a diagnosis is issued only once the compressor has reached a steady state, or that transient data is additionally collected and incorporated into the pre-training corpus. Extending the dataset to cover start-up and shutdown transients and continuously varying load profiles, and systematically evaluating the framework under such non-stationary conditions, constitutes an important direction for future work.

To place these limitations in perspective, future work should (i) validate the framework on public bearing and gearbox benchmark datasets and on compressors of different capacities and drive types; (ii) conduct a sensor-placement sensitivity analysis with multiple simultaneous measurement positions; (iii) quantify the impact of label noise in the support set on the prototype estimate; (iv) integrate an explicit uncertainty or rejection mechanism into the classification decision rule so that unreliable diagnoses caused by degraded input signals can be flagged rather than silently accepted; and (v) repeat the complete training pipeline across independently drawn group-wise (recording-level) splits, so as to quantify the sensitivity of the reported accuracies to the data partition (the multi-seed variability analysis is now reported in Section 4.4); (vi) upon acceptance, we will release the full training and inference code, and—subject to the commercial-confidentiality constraints stated in the Data Availability Statement—make the self-collected AC-compressor vibration dataset available to qualified researchers upon reasonable request, to support reproducibility and external validation.

## 5. Conclusions

This study demonstrates that the proposed Two-Stage Self-Supervised Meta-Learning (TS-SSML) framework provides an effective solution to the challenge of cross-operating-condition fault diagnosis for vibration-sensor-based industrial monitoring with limited labeled data. By integrating self-supervised contrastive pre-training with attention-weighted prototypical meta-learning, the framework learns fault representations that are transferable across operating conditions and achieves a robust few-shot classification capability. Experimental results on the self-collected AC compressor vibration dataset show that the proposed method achieves a 96.50% and 94.10% accuracy under the 5-shot setting on cross-condition transfer and incremental few-shot novel-class detection tasks, respectively, outperforming standard Prototypical Networks by 1.20% and 4.92%. Compared with the SCM-FD baseline, our method reduces the episodic meta-training time by 93.75% (Stage 2 few-shot adaptation only) while delivering a superior diagnostic performance, offering a practical technical reference for predictive maintenance in the refrigeration industry. We note that all reported results are obtained on a single 3.5 kW scroll-compressor test rig under two controlled load conditions with manually seeded faults (see Section 4.5); independent validation on public benchmark datasets and on field data from other compressor types remains necessary before broader conclusions can be drawn. The ablation further shows that the two components are complementary rather than interchangeable: the benefit of attention weighting is markedly larger under NT-Xent pre-training (+1.20 pp on T1, +4.72 pp on T2) than under supervised pre-training (+0.10 pp on T1, +0.70 pp on T2), which we attribute to the larger within-class dispersion that an instance-discrimination objective leaves uncorrected. We therefore describe the two stages as functionally coupled, and we do not characterize their contributions as simply additive; establishing the magnitude of this interaction more precisely would require completing the missing cell of the ablation and is left for future work.

With the continuous advancement of industrial sensing and edge computing technologies, self-supervised meta-learning approaches hold great promise for the intelligent operation and maintenance of rotating machinery. For future work, we will further validate the generalization capability of the framework across more types of industrial rotating equipment, and explore lightweight model adaptation to support real-time edge-end diagnosis scenarios.

## Figures and Tables

**Table 1:** CNN network architecture parameters.

Layer	Input Dimensions	Output Dimensions	Convolution Kernel	Pooling	Parameters
Input	(B, 1, 1024)	—	—	—	—
ConvBlock1	(B, 1, 1024)	(B, 64, 512)	3 × 1	2 × 1	256
ConvBlock2	(B, 64, 512)	(B, 64, 256)	3 × 1	2 × 1	12,416
ConvBlock3	(B, 64, 256)	(B, 64, 128)	3 × 1	2 × 1	12,416
ConvBlock4	(B, 64, 128)	(B, 64, 64)	3 × 1	2 × 1	12,416
Flatten	(B, 64, 64)	(B, 4096)	—	—	0
Fully connected layer	(B, 4096)	(B, 256)	—	—	1,048,832

**Table 2:** Distribution of compressor fault dataset categories.

Category Tags	Fault Condition	Severity	Number of Samples Under High-Pressure Conditions	Number of Samples Under Low-Pressure Conditions
0	Normal	—	1000	1000
1	Valve Fault	Minor (L)	980	975
2	Valve Fault	Moderate (M)	970	968
3	Valve Fault	Severe (H)	950	948
4	Piston Wear	Minor (L)	990	985
5	Piston Wear	Moderate (M)	975	971
6	Piston Wear	Severe (H)	945	940
7	Bearing Fault	Minor (L)	985	982
8	Bearing Fault	Moderate (M)	965	961
9	Bearing Fault	Severe (H)	940	937

**Table 3:** Hyperparameter settings for each method.

Method	Learning Rate	Optimizer	Encoder Structure	Training Epochs/Episodes	Other
CNN	0.001	Adam	1D-CNN (4 layers, 64 channels)	100 epochs	Training with the full dataset
CNN-FT	0.001/0.01	Adam	1D-CNN (4 layers, 64 channels)	150/50 epochs	Pre-training + Fine-tuning
CNN-MMD	0.001	Adam	1D-CNN (4 layers, 64 channels)	150 epochs	MMD regularization coefficient λ = 0.5
SCM-FD (MAML)	0.005/0.05	Adam	1D-CNN (4 layers, 64 channels)	1000 meta-epochs	Number of inner steps = 1 (5-shot) or 3 (1-shot)
ProtoNet	0.001	Adam	1D-CNN (4 layers, 64 channels)	10,000 episodes	*lr_d_ecay* = 0.95 (exponential decay)
RelationNet	0.001	Adam	1D-CNN (4 layers, 64 channels)	500 episodes	MSE loss Trainable similarity
AWPN (ours)	0.001	Adam	1D-CNN (4 layers, 64 channels)	E1 = 300/E2 = 150	τ = 0.5 (NT-Xent) Q = K shots

**Table 4:** Comparison of accuracy rates for each method in Task T1 (cross-condition transfer).

Method	1-Shot Accuracy (%)	5-Shot Accuracy (%)	Training Time (s)	Inference Time (ms)	Number of Parameters (K)
CNN	48.00 ± 1.40	71.80 ± 0.90	2.5	1.18	321
CNN-FT	48.00 ± 1.40	75.90 ± 0.90	2.5	3.45	321
CNN-MMD	—	81.35 ± 0.80	15.4	1.16	321
SCM-FD	87.40 ± 1.00	95.80 ± 0.80	720	5.65	321
Reptile	—	94.60 ± 0.60	1820	12.04	321
ProtoNet	87.69 ± 0.90	95.30 ± 0.40	41	0.29	160
RelationNet	—	92.34 ± 0.50	304	0.35	1339
AWPN (ours)	—	96.50 ± 0.40	45	0.28	165

Note: All accuracies in this table are mean ± standard deviation over K = 5 independent random seeds (2021–2025), each seed averaged over 600 test episodes. Confidence intervals are calculated based on the standard error (×1.96) of 600 random episodes; “—” indicates that the configuration was not included in the comparative experiments.

**Table 5:** Comparison of accuracy rates for various methods in Task T2 (incremental few-shot novel-class detection). Note: the training time listed for AWPN is the Stage 2 meta-training time only; Stage 1 NT-Xent self-supervised pre-training takes ≈ 22,800 s on unlabeled data (a one-time, reusable cost).

Method	1-Shot Accuracy (%)	5-Shot Accuracy (%)	Training Time (s)	Inference Time (ms)
CNN-FT	68.00 ± 1.58	82.50 ± 1.20	—	4.68
SCM-FD	77.50 ± 1.32	91.95 ± 0.90	340	4.46
ProtoNet	77.25 ± 1.21	89.18 ± 0.90	—	0.10
RelationNet	—	93.22 ± 0.80	275	0.19
AWPN (ours)	—	94.10 ± 0.50	52	0.17

Note: accuracies are mean ± std over K = 5 seeds.

**Table 6:** Results of the ablation experiments for each AWPN module.

Variant	Pre-Training	Prototype Weighting	T1 Acc. (%)	T2 Acc. (%)	Δ vs. Equal Weights, T1 (pp)	Δ vs. Equal Weights, T2 (pp)
(a) No pre-training + equal weights	Random initialization	Equal	94.80 ± 0.40	90.50 ± 0.50	—	—
(b) Supervised pre-training + equal weights	Supervised learning	Equal	95.80 ± 0.40	92.30 ± 0.53	—	—
(c) Supervised pre-training + attention weighting	Supervised learning	Attention-weighted	95.90 ± 0.45	93.00 ± 0.51	+0.10	+0.70
(d) Self-supervised pre-training + equal weights	NT-Xent (label-free)	Equal	95.30 ± 0.42	89.38 ± 0.51	—	—
(e) Self-supervised pre-training + attention weighting (AWPN, ours)	NT-Xent (label-free)	Attention-weighted	96.50 ± 0.43	94.10 ± 0.71	+1.20	+4.72

Note: all accuracies are mean ± standard deviation over K = 5 independent random seeds, each seed averaged over 600 test episodes. Rows are arranged so that the two factors can be read off separately: rows (b) and (d) each differ from row (a) only in the pre-training mode (prototype weighting held equal), and each of them differs from the row immediately below—(c) and (e), respectively—only in the prototype weighting scheme. The last two columns therefore isolate the contribution of attention weighting within each pre-training mode. A “no pre-training + attention weighting” configuration is not included, so the random-initialization level is not fully crossed with the weighting factor; see the caveats in Section 4.3.1.

## Data Availability

The industrial vibration dataset used in this study contains proprietary production data and is not publicly available due to commercial confidentiality.

## Abbreviations

The following abbreviations are used in this manuscript:

AC	air conditioning
AE	autoencoder
AWP	attention weighting module
AWPN	attention-weighted prototypical network
BN	batch normalization
CNN	convolutional neural network
CPU	central processing unit
CUDA	compute unified device architecture
ELM	extreme learning machine
GPU	graphics processing unit
MAML	model-agnostic meta-learning
MLP	multilayer perceptron
MMD	maximum mean discrepancy
NT-Xent	normalized temperature-scaled cross-entropy
ProtoNet	prototypical network
RAM	random access memory
ReLU	rectified linear unit
RF	random forest
RNN	recurrent neural network
SCM-FD	sparse coding-based fault diagnosis
SVM	support vector machine
t-SNE	t-distributed stochastic neighbor embedding
TS-SSML	two-stage self-supervised meta-learning
VRAM	video random access memory
Nomenclature	
Symbol	Definition
$C_{\mathrm{base}}$	set of base fault classes covered by the training set
$C_{\mathrm{novel}}$	set of novel fault classes from which the few-shot tasks are constructed
$D_{\mathrm{train}}$	training set, which covers only the base classes
$D_{s},\;D_{t}$	source domain (high-pressure condition) and target domain (low-pressure condition)
N, K	number of classes (ways) and number of labeled support samples per class (shots) in a few-shot task
T	an N-way K-shot few-shot classification task, *T* = (*S*, *Q*)
S, Q	support set and query set of a few-shot task
$N_{s},\;N_{t}$	number of labeled samples available in the source and target domains
$x_{i}^{s},y_{i}^{s}$	i-th labeled vibration signal and its label in the source domain
x	augmented signal obtained from the raw signal x
σ, ε	standard deviation of the injected Gaussian noise and the corresponding Gaussian random variable
A	random amplitude-scaling factor drawn from a uniform distribution
Δ, L	random time-shift offset and the signal length
p	inversion probability of the signal-inversion augmentation (p = 0.5)
$f_{\theta }(\cdot )$	1D-CNN feature encoder parameterized by θ, mapping the input space X to the feature space Z
z	256-dimensional feature vector produced by the encoder
$g_{\varphi }(\cdot )$	two-layer MLP projection head parameterized by φ (used in Stage 1 only)
$z_{i}^{1},\;z_{i}^{2}$	projected features of the two augmented views of sample i
τ	temperature parameter of the NT-Xent loss
B	number of samples in a contrastive pre-training batch
sim(·,·)	cosine similarity between a pair of projected features
$l_{j(i)}^{i}$$,L_{\mathrm{SSL}}$	NT-Xent loss of the positive pair of sample i and its batch average
$p_{c}$	prototype (class representative) of class c
d(·,·)	Euclidean distance used by the ProtoNet classification head
$e_{i}^{c}{,\;\alpha }_{i}^{c}$	attention score and normalized attention weight of support sample i of class c
W, b, w	projection matrix, bias, and attention vector of the bilinear attention module
$d_{a}$	dimension of the attention hidden layer (set to 64)
$p_{\psi }(c|x_{q})$	classification probability of a query sample predicted by the AWPN head
$y_{c}$	one-hot ground-truth label of class c
$L_{\mathrm{AWPN}}$	cross-entropy loss of the attention-weighted prototype network
E1, E2	Stage 1 pre-training epochs and Stage 2 meta-training episodes
Task T1/T2	evaluation tasks: cross-condition transfer task and incremental novel-class detection task
